# DyNDG: Identifying Leukemia-related Genes Based on Time-series Dynamic Network by Integrating Differential Genes

**DOI:** 10.1093/gpbjnl/qzaf037

**Published:** 2025-04-29

**Authors:** Jin A, Ju Xiang, Xiangmao Meng, Yue Sheng, Hongling Peng, Min Li

**Affiliations:** School of Computer Science and Engineering, Central South University, Changsha 410083, China; School of Computer and Communication Engineering, Changsha University of Science & Technology, Changsha 410114, China; School of Computer Science & School of Cyberspace Science, Xiangtan University, Xiangtan 411105, China; Department of Hematology, The Second Xiangya Hospital, Central South University, Changsha 410011, China; Hunan Engineering Research Center of Cell Immunotherapy for Hematopoietic Malignancies, Changsha 410011, China; Department of Hematology, The Second Xiangya Hospital, Central South University, Changsha 410011, China; Hunan Engineering Research Center of Cell Immunotherapy for Hematopoietic Malignancies, Changsha 410011, China; School of Computer Science and Engineering, Central South University, Changsha 410083, China

**Keywords:** Leukemia, Dynamic network, Random walk, Differentially expressed gene, Disease gene prediction

## Abstract

Leukemia is a malignant disease characterized by progressive accumulation with high morbidity and mortality rates, and investigating its disease genes is crucial for understanding its etiology and pathogenesis. Network propagation methods have emerged and been widely employed in disease gene prediction, but most of them focus on static biological networks, which hinders their applicability and effectiveness in the study of progressive diseases. Moreover, there is currently a lack of special algorithms for the identification of leukemia disease genes. Here, we proposed a novel Dynamic Network-based model integrating Differentially expressed Genes (DyNDG) to identify leukemia-related genes. Initially, we constructed a time-series dynamic network to model the development trajectory of leukemia. Then, we built a background–temporal multilayer network by integrating both the dynamic network and the static background network, which was initialized with differentially expressed genes at each stage. To quantify the associations between genes and leukemia, we extended a random walk process to the background–temporal multilayer network. The results demonstrate that DyNDG achieves superior accuracy compared to several state-of-the-art methods. Moreover, after excluding housekeeping genes, DyNDG yields a set of promising candidate genes associated with leukemia progression or potential biomarkers, indicating the value of dynamic network information in identifying leukemia-related genes. The implementation of DyNDG is available at both https://ngdc.cncb.ac.cn/biocode/tool/BT7617 and https://github.com/CSUBioGroup/DyNDG.

## Graphical abstract

**Figure:**
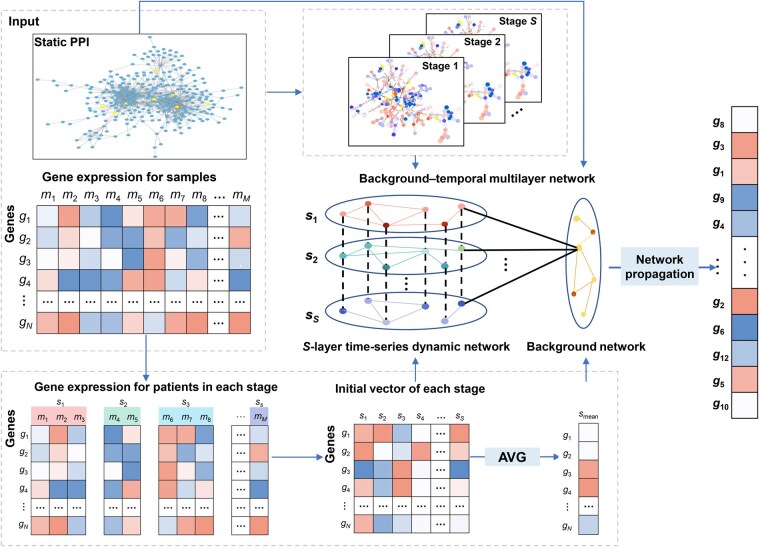

## Introduction

Blood disorders often give rise to pathological conditions that extend beyond the blood and may lead to dysfunction in other vital organs, posing a threat to human health. People may suffer from various types of blood conditions and blood cancers. Common blood disorders include anemia (such as hemolytic anemia), bleeding disorders (such as hemophilia and blood clots), and blood cancers (such as leukemia, lymphoma, and myeloma). Leukemia, specifically, is a malignant blood disease characterized by the overproduction of immature or abnormal white blood cells, which eventually suppresses the production of normal blood cells and causes symptoms associated with cytopenia. According to the updated global cancer statistics 2024 released by the World Health Organization’s International Agency for Research on Cancer (IARC), there were 9.7 million cancer deaths worldwide, including over 305,033 deaths from leukemia, while the number of leukemia deaths in China reached 50,100 [1]. The occurrence and development of leukemia are closely linked to the mutation and abnormal expression of genes that regulate cell growth in the bone marrow [2], although genetic, behavioral, and environmental factors collectively contribute to leukemia [3]. Therefore, investigating the causative genes of leukemia holds significant importance to uncover its pathogenesis. Identifying the causative genes of leukemia is an extremely challenging task due to the pleiotropy of genes, the genetic heterogeneity of diseases, and the limited number of study subjects [4–6].

With the continuous explosion of biological data, computational methods for predicting potential pathogenic genes have emerged, playing a significant role in disease research, prevention, and detection [7]. The functions of biomolecular components in cells are often interdependent rather than acting independently. Diseases typically arise from disruptions in a complex biomolecular network caused by genetic variants, pathogens, and epigenetic changes, rather than solely from abnormal expression of individual genes [8]. Therefore, numerous classical disease–gene prediction methods based on biomolecular networks have been proposed [9–16], among which network propagation has gained popularity due to its remarkable performance [17–21].

However, most traditional network-based methods are based on static biomolecular network structures. In contrast, cellular systems are highly dynamic and respond dynamically to external changes in different time points, spaces, and conditions [22]. Thus, the relationships between biomolecules are constantly changing in response to time and conditions, and the molecular mechanisms underlying disease onset and progression are closely related to this dynamic nature. Traditional static networks lose the dynamic information due to their highly averaged and idealized structures, hindering the further advancement of network-based methods [23,24]. Some researchers have already shifted their attention from static biological networks to dynamic biological networks [25]. The key challenge in constructing dynamic biological networks is how to determine the dynamic features of biomolecule expression at different time points. Initially, de Lichtenberg et al. [26] proposed that persistently expressed proteins are not dynamic, while cyclically expressed proteins are only expressed at their highest levels in the expression cycle. Hegde et al. [27] suggested using the average expression value of each region in the network as the threshold for judging which proteins are actually expressed in that region. Tang et al. [28] discovered that periodically expressed genes often exhibit peak expression levels greater than a fixed constant by studying the dynamic protein interaction network in yeast cells. In contrast to the former fixed threshold method, Zhang et al. [29] introduced a *k*-sigma method based on the 3-sigma rule by designing an activity threshold for each gene based on its dynamic expression. The development of dynamic protein network construction methods, which simulate the operational rules of real biological systems, effectively overcomes the limitations of static protein network-based analysis methods and plays a significant role in protein complex identification [30,31], protein function prediction [32,33], and biomarker identification [34,35]. Numerous studies have demonstrated that dynamic protein networks can yield superior results in various related issues since they can better reflect the dynamic properties of biological processes over time and in response to external environments [36,37]. It is necessary to simulate the dynamic changes of biomolecular networks during disease progression to reveal the associations between diseases and genes.

Therefore, we proposed the Dynamic Network-based model integrating Differentially Expressed Genes (DyNDG) for identifying leukemia-related genes. We took three common leukemias: chronic myeloid leukemia (CML), chronic lymphocytic leukemia (CLL), and acute myeloid leukemia (AML) as research subjects. For each leukemia, DyNDG first generates a time-series dynamic network using expression data over stages of disease development, and then constructs a background–temporal multilayer network by integrating both the static network structure information and the dynamic information of leukemia development. Moreover, initialized by differentially expressed genes (DEGs), DyNDG extends a random walk process into the multilayer network to extract scores of leukemia-related genes. Results on the three types of leukemia and three control sets demonstrate that considering the time-series dynamics of leukemia development through DyNDG significantly enhances the accuracy of prioritizing leukemia-related genes compared to popular methods. Directly using predicted leukemia-related genes as drug targets may lead to interference and toxic side effects on normal cells. In order to minimize and avoid this possible harm, we selected higher-ranked genes from the predicted candidate gene list, excluded housekeeping genes from them, and yielded a set of promising candidate genes associated with leukemia. Through the integration of multiple analysis methods, we aimed to further understand the functions and regulatory mechanisms of these genes in the development of leukemia, providing valuable references and assistance for researchers and doctors.

## Method

### Datasets

#### Time-series expression data for leukemia

Gene Expression Omnibus (GEO) database is a public functional genomics data repository, which includes different gene expression datasets under different designs and conditions. Time-series expression data for CML, CLL, and AML were obtained from the GEO database (https://www.ncbi.nlm.nih.gov/geo/) (GEO: GSE47927, GSE2403, and GSE122917, respectively). We preprocessed the data in the following three steps: (1) mapping of gene IDs and gene symbols; (2) filtering genes encoding proteins; and (3) cleaning the data. Subsequently, the expression data were subjected to pathological analysis to classify the stages of leukemia development.

The development of leukemia is a multi-factor and multi-step cancerous process. Leukemia is generally categorized into two types: acute leukemia and chronic leukemia, based on the differentiation and maturation status of leukemic cells and the natural progression of the disease [38]. AML, as a typical representative of acute leukemia, progresses rapidly and its disease stages are temporarily described as untreated, in remission, refractory, or recurrent [39], because there is currently no standard disease staging system for it. Chronic leukemia develops slowly and has a natural course of several years. In particular, CLL follows the Rai system, which classifies CLL into “low-risk group (stage 0), intermediate-risk group (stages I and II), and high-risk group (stages III and IV)” [40]. CML is categorized into three disease phases: chronic phase (CP), accelerated phase (AP), and blast phase (BP) [41]. **Table 1** provides detailed information on the time-series staging sample statistics for GSE47927, GSE2403, and GSE122917, based on their corresponding staging systems.

**Table 1:** Details of time-series staging sample statistics

Disease	Data	Period	Symbol	No. of samples
CML	GSE47927	Normal	Normal	15
		Chronic phase	CP	24
		Accelerated phase	AP	18
		Blast phase	BP	10
CLL	GSE2403	Low-risk	Rai 0	8
		Intermediate-risk	Rai I and Rai II	7
		High-risk	Rai III and Rai IV	6
AML	GSE122917	Normal	0	3
		Untreated	1	3
		Recurrent	2	3

*Note*: CLL, chronic lymphocytic leukemia; CML, chronic myeloid leukemia; AML, acute myeloid leukemia.

#### Biological networks

STRING is a database that covers the largest number of species and contains the most extensive information on protein–protein interactions (PPIs). It collects and integrates known and predicted protein–protein association data from a variety of sources, including automated text mining of scientific literature, computational interaction predictions based on coexpression analyses, interaction experiments, and known complexes/pathways from curated sources [42]. HumanNet is an integrated human gene network database for disease research. It was constructed by incorporating new data types, expanding data sources, and utilizing improved network inference algorithms [43]. Due to the rich PPIs collected in STRING (https://cn.string-db.org/) and the focus of HumanNet (https://staging2.inetbio.org/humannetv3/) on disease research, we chose to use data from the two databases to construct the static PPI networks, respectively. Further details regarding the static PPI networks can be found in Table S1.

#### Disease genes for leukemia

Known leukemia-related genes were obtained from the MalaCards database (https://www.malacards.org/) (MCID: LKM071, LKM063, and LKM061) [44], which is an integrated database of human diseases and their annotations. There are 527 AML-related genes, 204 CML-related genes, and 339 CLL-related genes collected from the MalaCards database. We filtered these genes using protein-coding genes curated from the Human Genome Organization (HUGO) Gene Nomenclature Committee (HGNC) database (https://housekeeping.unicamp.br/?download) [45].

### Framework of DyNDG

**Figure 1:**
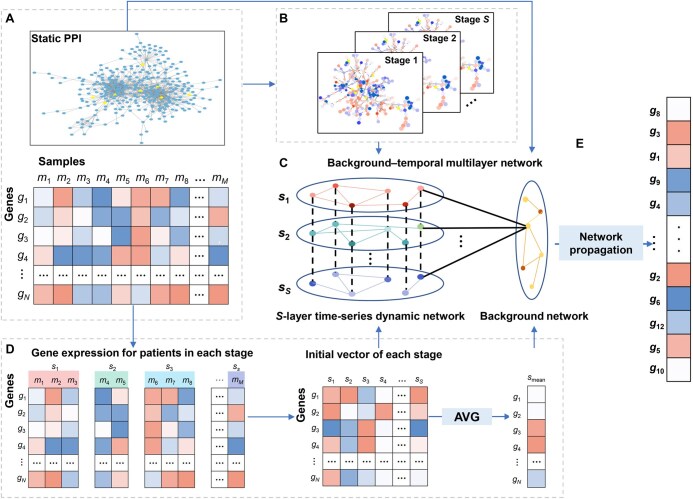
The schematic diagram of DyNDG **A**. The original inputs to DyNDG. **B**. The *S*-layer time-series dynamic network. **C**. The background–temporal multilayer biological network. The initial probability vector for each stage is applied to corresponding gene nodes in the corresponding-stage network layer. Smean is applied to corresponding gene nodes in the background network layer. **D**. Obtaining the initial probability vector of gene nodes for each stage through differential expressed gene analysis from time-series gene expression data of patients. Smean means taking the arithmetic mean of the initial probability vectors across all stages. Smean is used as the initial probability vector of gene nodes in the background network layer. **E**. The vector of scores for leukemia-related genes is obtained as the output of network propagation on the background–temporal multilayer biological network. DyNDG, Dynamic Network-based model integrating Differentially Expressed Genes; PPI, protein–protein interaction; AVG, average.

Here, we proposed a dynamic network-based model, DyNDG, for predicting leukemia-related genes. DyNDG involves constructing the time-series dynamic biological networks and the background–temporal multilayer biological network, followed by network propagation on the multilayer biological network (**Figure 1**). The original inputs consist of a gene expression matrix and a static PPI network (Figure 1A). After constructing the time-series dynamic network (Figure 1B), the S-layer time-series dynamic network and the background network are combined to form the background–temporal multilayer biological network (Figure 1C). The initial probability vector of genes in the multilayer network is derived through differential gene expression analysis (see Figure 1D and the section “Network propagation on background–temporal multilayer biological network” for details). Subsequently, a network propagation process on the background–temporal multilayer biological network, which is initialized by DEGs, is performed to generate the predictive scores for leukemia-related genes (Figure 1E).

### Construction of time-series dynamic networks

The disease progression of leukemia involves multiple stages, and the biological networks associated with leukemia undergo dynamic changes throughout these stages. The gene expression data capturing the progression of leukemia were obtained by investigating patient populations at different stages of the disease, which can provide valuable insights into the dynamic gene changes that occur as leukemia progresses. The biological network constructed for each stage encompasses the gene interaction information from all patients within that stage. Given the inherent noise in gene expression data and the diverse expression patterns across genes, we employed the *k*-sigma method to calculate the active probability of each gene across various patient samples [29]. This approach effectively distinguishes the active level of genes in each patient sample when constructing the biological network for each disease stage. The gene expression matrix $G^{N\times M}$ contains expression profiles of M patient samples from S disease stages, where N is the number of genes. For each patient sample m, $G_{m}(g_{n})$ denotes the gene expression value of gene $g_{n}$ in this patient. For all M patient samples, define $\bar{G(g_{n})}$ and $\sigma (g_{n})$ as the algorithmic mean and standard deviation of gene expression values for $g_{n}$, respectively. We calculated k-sigma threshold (where k represents the number of sigma) of each gene $g_{n}\;$by:


#(1) $${\mathrm{Active}\_Th}_{k}(g_{n})=\bar{G(g_{n})}+k\cdot \sigma (g_{n})\cdot (1-\frac{1}{1+\sigma^{2}(g_{n})}),\;k=1,\;2,\;\mathrm{or}\;3\;$$

#(2) G(gn)¯=∑m = 1MGm(gn)M

#(3) σ2(gn)=∑m = 1M(Gm(gn)-G(gn)¯)2M−1

${\mathrm{Active}\_Th}_{k}(g_{n})\;$ represents k active threshold of $g_{n}$. The active probability of a protein corresponding to gene $g_{n}$ in the patient sample m is calculated as follows [29]:


#(4) Active_Prm(gn)={0.99if Gm(gn)≥Active_Th3(gn)0.95if Active_Th3(gn)>Gm(gn)≥Active_Th2(gn)0.680if Active_Th2(gn)>Gm(gn)≥Active_Th1(gn)if Gm(gn)<Active_Th1(gn)

In general, the active probability value of a protein can be used as a measure of its active level in a patient sample. In order to construct the time-series dynamic network, we initially constructed the PPI network$\;{\mathrm{Net}}_{m}(V_{m}^{1\times N},\;E_{m},\;{AP}_{m}^{1\times N},\;A_{m})$ for each patient sample m; $V_{m}^{1\times N}=\{v_{1},\ldots ,v_{i},\ldots ,v_{j},\ldots ,v_{N}\}$ is the set of N gene/protein nodes;${\;E}_{m}$ is the set of interactions; ${AP}_{m}^{1\times N}$ is the active probability vector of N gene/protein nodes; $A_{m}$ represents the adjacency matrix which represents the confidence of the interactions between genes. Reweight $A_{m}$ to $A_{m}^{\prime }\;$using ${AP}_{m}^{1\times N}$ as follows:


#(5) Am'[vi,vj]=APm[vi]×Am[vi,vj]×APm[vj]

For the sake of concise and compact expression, a calculation method called “Broadcasting Multiplication” can be employed to describe the reweighting operation. The calculation process of Equation 5 can be found in section 1 of File S1.

Divide M samples into S leukemia stages, and the number of samples in each stage s is $M_{s}$. The adjacency matrix $A^{\left[s\right]}(s=1,\ldots ,S)$ of leukemia stage s can be calculated as follows:


#(6) A[s]=∑m = 1MsAm'Ms

**Figure 2:**
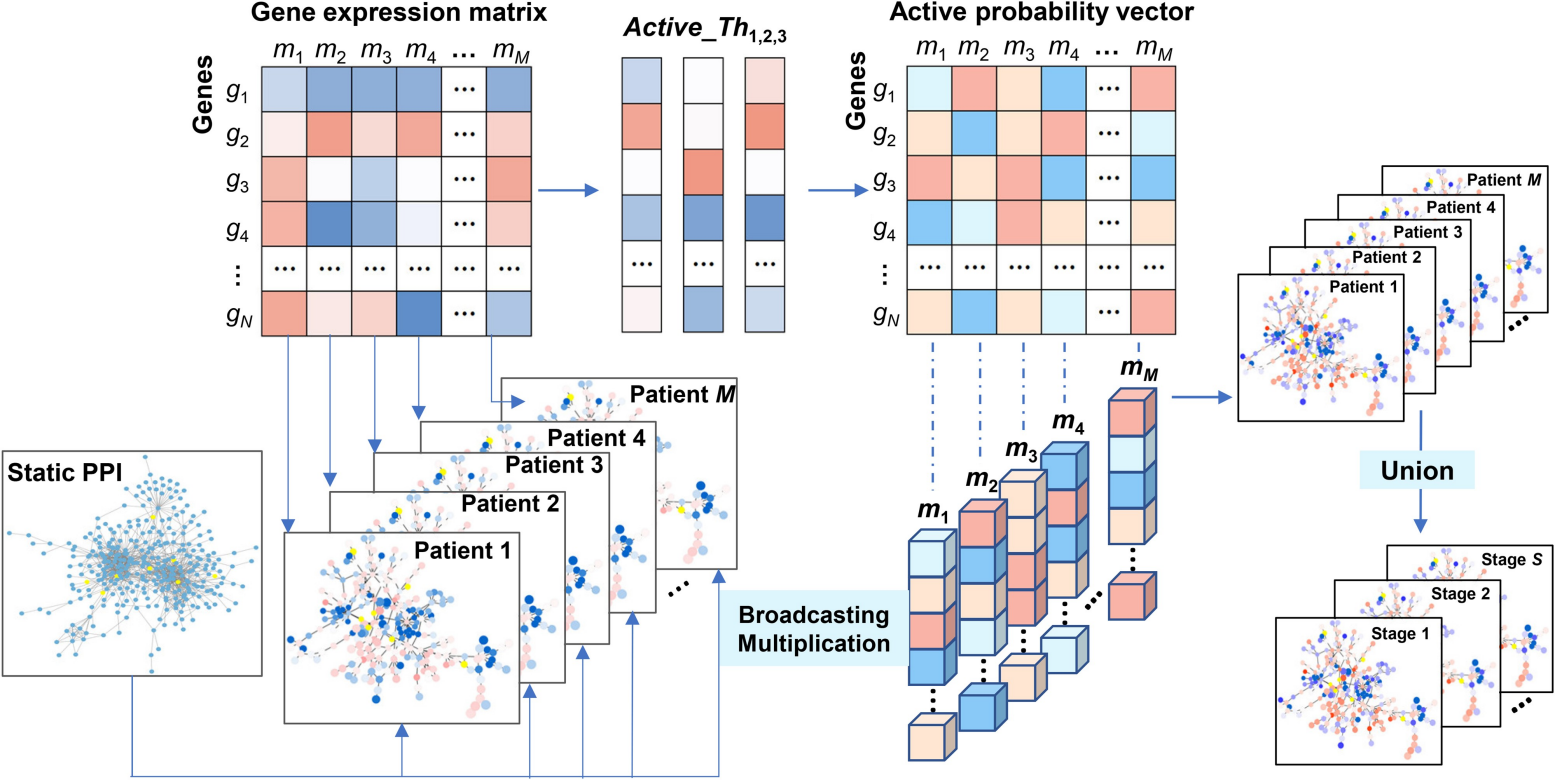
The schematic diagram of time-series dynamic network construction The process of constructing time-series dynamic network. Inputs consist of a gene expression matrix and a static PPI network. Union indicates network union of patient samples at the same stage.

Finally, we constructed the time-series dynamic networks based on the adjacency matrix $A^{\left[s\right]}$ of each leukemia stage. **Figure 2** shows the process of building the time-series dynamic networks. Firstly, the k-sigma threshold for each gene $g_{n}$ is calculated from the gene expression matrix. The active probability vector of proteins corresponding to genes is obtained by using this threshold. By integrating the gene expression matrix and the static PPI network, we obtained the initial network for each patient sample. Further, new networks of patient samples are obtained by reweighting the edges with the active probabilities of the two corresponding genes using “Broadcasting Multiplication”. The time-series dynamic networks corresponding to multiple leukemia stages are obtained by taking the “Union” of the patient sample networks. Note that the networks for all stages have the same number of genes, but the genes in different networks may be interconnected with different weights.

### Construction of background–temporal multilayer biological network

The comprehensiveness and richness of biological networks significantly impact the ability to predict leukemia genes. To capture more realistic network changes throughout the course of leukemia, building upon the time-series dynamic networks simulating the dynamic alterations in biological networks, we proposed a background–temporal multilayer biological network framework. This framework integrates the dynamic networks and the static network into a multilayer network, effectively amalgamating information from both static and dynamic networks. The multilayer network is constructed by connecting shared gene nodes across different network layers, for jumping to a different layer through the same node when conducting propagation on it. The specific steps are as follows. Firstly, we constructed a S-layer temporal dynamic network by connecting the S time-series dynamic network layers of neighboring disease stages through shared nodes. Secondly, we connected the background network layer constructed by the static PPI network with each layer of the S-layer dynamic network by linking the corresponding gene counterparts. In the end, we obtained a connected multilayer network ${\mathrm{Net}}_{\mathrm{multi}}=(V_{\mathrm{multi}},\;{\;E}_{\mathrm{multi}},\;A_{\mathrm{multi}})$ with L(=S+1) layers, where S is the number of layers in the aforementioned dynamic network; $A_{\mathrm{multi}}=\{A^{\left[1\right]},{\ldots ,A}^{\left[S\right]},A^{\left[L\right]}\}$ is the set of adjacency matrixes for the L layers, and $A^{\left[L\right]}$ is the adjacency matrix of static PPI network; $V_{\mathrm{multi}}=\{v_{i}^{\left[l\right]}\;|\;i=1∼N,\;l=1∼L\}$ is the set of nodes (note that each gene has a counterpart node in each network layer); $E_{\mathrm{multi}}=\{\mathrm{edge}(v_{i}^{\left[l\right]},\;v_{j}^{\left[l\right]})\;|\;i=1∼N-1,\;j=i+1∼N,\;l=1∼L,{\;A}^{\left[l\right]}[v_{i}^{\left[l\right]},\;v_{j}^{\left[l\right]}]\;\neq \;0\}\cup \{\mathrm{edge}(v_{i}^{\left[\alpha \right]},v_{i}^{\left[\beta \right]})|i=1∼\;N,\;\alpha =1∼S-1,\;\beta =\alpha +1\}\cup \{\mathrm{edge}(v_{i}^{\left[\alpha \right]},v_{i}^{\left[\beta \right]})\;|\;i=1∼N,\;\alpha =1∼S,\;\beta =L\}$ is the set of interlayer connections and intralayer PPIs. Note that all the edges here are undirected. Complex information about leukemia progression flows through edges in the multi-layer network. The background–temporal multilayer biological network consists of two parts: the static network and the time-series dynamic network. The relevant details of the background–temporal multilayer biological network are presented in Table S2.

### Network propagation on background–temporal multilayer biological network

To identify leukemia-related genes, we applied a network propagation process on the background–temporal multilayer biological network ${\mathrm{Net}}_{\mathrm{multi}}$. During the walking process, the particles in the ${\mathrm{Net}}_{\mathrm{multi}}$ either restart with a certain probability, or walk along a randomly selected (intra-layer or inter-layer) edge to a neighboring node (corresponding one of two possible actions: walk within the same layer, or jump to the counterparts of the same node in a different layer). Finally, as the random walk process concludes after a certain period, the scoring value of each node in the network will be obtained. During the random walk process, the scoring vector $P_{t+1}$ of the nodes at step t+1 can be obtained as follows:


#(7) Pt+1=(1-γ)TLcPt+γPRS

where γ∈(0,1) is the probability of restart, $T_{L}^{c}$ is the column-normalized transition matrix of the multilayer network, $P_{RS}\in R^{NL\times 1}$ represents the initial scoring vector of NL nodes in the L-layer network, and $P_{t}\in R^{NL\times 1}$ represents the scoring vector of NL nodes at step t.

Different stages of disease development are often accompanied by changes in gene expression levels and dynamic alterations in the network structure of gene interactions. However, most existing disease gene prediction methods do not take these biological phenomena into account. To address this, we proposed incorporating differential information during leukemia development as prior knowledge in the network propagation model. Specifically, for the gene expression matrix divided into S disease stages, we conducted differential gene expression analysis between each stage and the others by using the limma [46] R package to obtain the T-statistic values of the genes in the corresponding layer of each stage. The absolute value of these T-statistic values is used to determine the initial scoring vector of genes, as they contain valuable information that effectively distinguishes each stage from the others. For the temporal multilayer network, we use the absolute T-statistics of the genes calculated in each of the *S* stages as the initial probability for each stage, thus incorporating stage-specific differential gene expression information. For the background network, we calculated the average of the aforementioned gene scores obtained from the differential analysis across all stages as the initial scores for each gene. By incorporating the T-statistic values into the background–temporal multilayer biological network, we can enhance the network with more comprehensive stage-specific information. As a result, we got the initial scoring vector $P_{RS}\in R^{NL\times 1}$ in the L-layer network, which incorporates differential information of gene expression during disease progression as prior knowledge into the network.

Then, we constructed the NL×NL transition matrix $T_{L}$ of the L-layer network by


#(8) TL=[(1-δ)BδJTδJTS]

where δ∈[0, 1], $e={\left(1,1,\ldots \ldots ,1\right)}^{T}\in R^{S\times 1}$, $J=\frac{1}{S}e⊗I$, and I is a N×N identity matrix. Here, *B* is the adjacency matrix of the background network, δ controls the probability of jumping between two types of network layers, and J is the inter-network transition matrix that controls the jumping from the background network layer to each layer of the time-series dynamic network. Let $T_{S}$ be the NS×NS transition matrix of the S-layer temporal dynamic network by


#(9) TS={(1-μ)A[1]μI0⋯0μI(1-μ)A[2]⋱⋱⋮0⋱⋱⋱0⋮⋱⋱(1-μ)A[S−1]μI0…0μI(1-μ)A[S]}

where μ∈[0, 1] controls the transition probability between the temporal dynamic network layers and $A^{\left[s\right]}\;(s=1∼S)$ represents the adjacency matrix of each layer of the S-layer dynamic network. The matrix $T_{S}$ controls the inter-layer and intra-layer jumps of the S-layer temporal dynamic network. We obtained $T_{L}^{c}$ by column normalization of matrix $T_{L}$. The example and parameter description of Equation 9 can be found in section 2 of File S1.

We plunged the column-normalized matrix $T_{L}^{c}$ and initial scoring vector $P_{RS}$ into the aforementioned iterative Equation 7, which is iteratively updated until the difference between $P_{t}$ and $P_{t+1}$ falls below 10^−6^. Upon convergence, gene nodes in the background–temporal multilayer network are assigned a steady-state scoring vector $P_{\infty }$. In the background network, the steady-state scoring vector $P_{\infty }^{B}$ gives a measure of how strongly a gene is associated with leukemia. If $P_{\infty }^{B}(v_{i})>P_{\infty }^{B}(v_{j})$, the gene corresponding to node $v_{i}$ is more closely related to leukemia. Then genes are sorted in descending order based on $P_{\infty }^{B}$, generating a ranking list of leukemia-related genes. Therefore, the higher-ranked genes can be considered as promising genes related to leukemia.

### Evaluation methods

#### Evaluation strategy and metrics

We conducted a systematic evaluation of the proposed DyNDG model, which involves constructing a background–temporal multilayer network and predicting leukemia-related genes on this network. The set of candidate genes consists of both a test gene set and a control gene set. The test set is composed of known disease genes. We considered three different schemes for constructing the control set. (1) Artificial Linked Interval Control Set (ALI): In this scheme, resembling linkage analysis or association studies, we selected 99 control genes for each test gene from the genes located closest to the test gene on the same chromosome. (2) Randomized Control Set (RC): This scheme mimics the scenario in exome sequencing studies. We randomly selected 99 control genes for each test gene from the entire genome. (3) Whole Genomic Control Set (WG): In this scheme, all genes in the network, excluding the known disease genes, were considered as control genes. For each leukemia, let ${\mathrm{Set}}_{t}$ denote the test gene set, let ${\mathrm{Set}}_{c}$ denote the control gene set, while $\mathrm{Set}=\{{\mathrm{Set}}_{t},{\mathrm{Set}}_{c}\}$ represents the set of candidate genes. Combining the ranking list of genes obtained by DyNDG with Set can obtain the top *k* ranking list $R_{k}$ of candidate genes. We can quantitatively calculate four common metrics, *i.e.*, Topk_Precision, Topk_Recall, area under the receiver operating characteristic curve (AUROC), and area under the precision–recall curve (AUPRC), to evaluate the proposed method.

#### Comparison algorithms

Network-based methods have gained significant popularity in predicting disease genes and have demonstrated remarkable prediction results. To compare the performance, we considered two categories of network-based disease gene prediction algorithms (Table S3). For each category, we selected representative algorithms: (1) algorithms based on the single network: Random Walk with Restart (RWR) [19]; (2) algorithms based on multiple networks: methods utilizing multiple independent networks such as Random Walk with Restart integrating the Discounted Rating System (RWRDRS) [47], and methods based on multiple interconnected networks like Random Walk with Restart on the Merged Graph (RWRMG) [18] and Random Walk with Restart on Multiplex Graphs (RWRMP) [20]. Furthermore, it is a common strategy to leverage the differences in gene expression levels between normal and disease stages in predicting disease genes. Hence, we also included two representative algorithms: (1) the T-test method, which generates disease association measures similar to network-based methods but without utilizing network information; and (2) the Disease-Specific Network Enhancement Prioritization (DiSNEP) method, which enhances a general gene network into a disease-specific network and then prioritizes gene associations on the enhanced network [48].

### Functional enrichment analysis

Functional enrichment analysis is a technique widely used in bioinformatics to identify biological functions or processes that are overrepresented in a given set of genes or proteins. This analysis helps in understanding the biological significance of a gene list or a set of DEGs by linking them to known biological functions, pathways, or processes. We used the clusterProfiler R package [49] to perform Gene Ontology (GO) enrichment analysis and Kyoto Encyclopedia of Genes and Genomes (KEGG) pathway enrichment analysis on the top 1% ranked genes in the predicted leukemia-related candidate gene list to investigate their associations with leukemia-related functions and metabolic pathways.

## Results and discussion

### DyNDG greatly improves predictive ability

To evaluate the performance of DyNDG quantitatively, we generated the set of leukemia-related candidate genes by the decreasing order of the predictive scores, and the evaluation of DyNDG was conducted by four common metrics (*i.e.*, Topk_Precision, Topk_Recall, AUROC, and AUPRC), along with three kinds of the control sets: ALI, RC, and WG.

**Figure 3:**
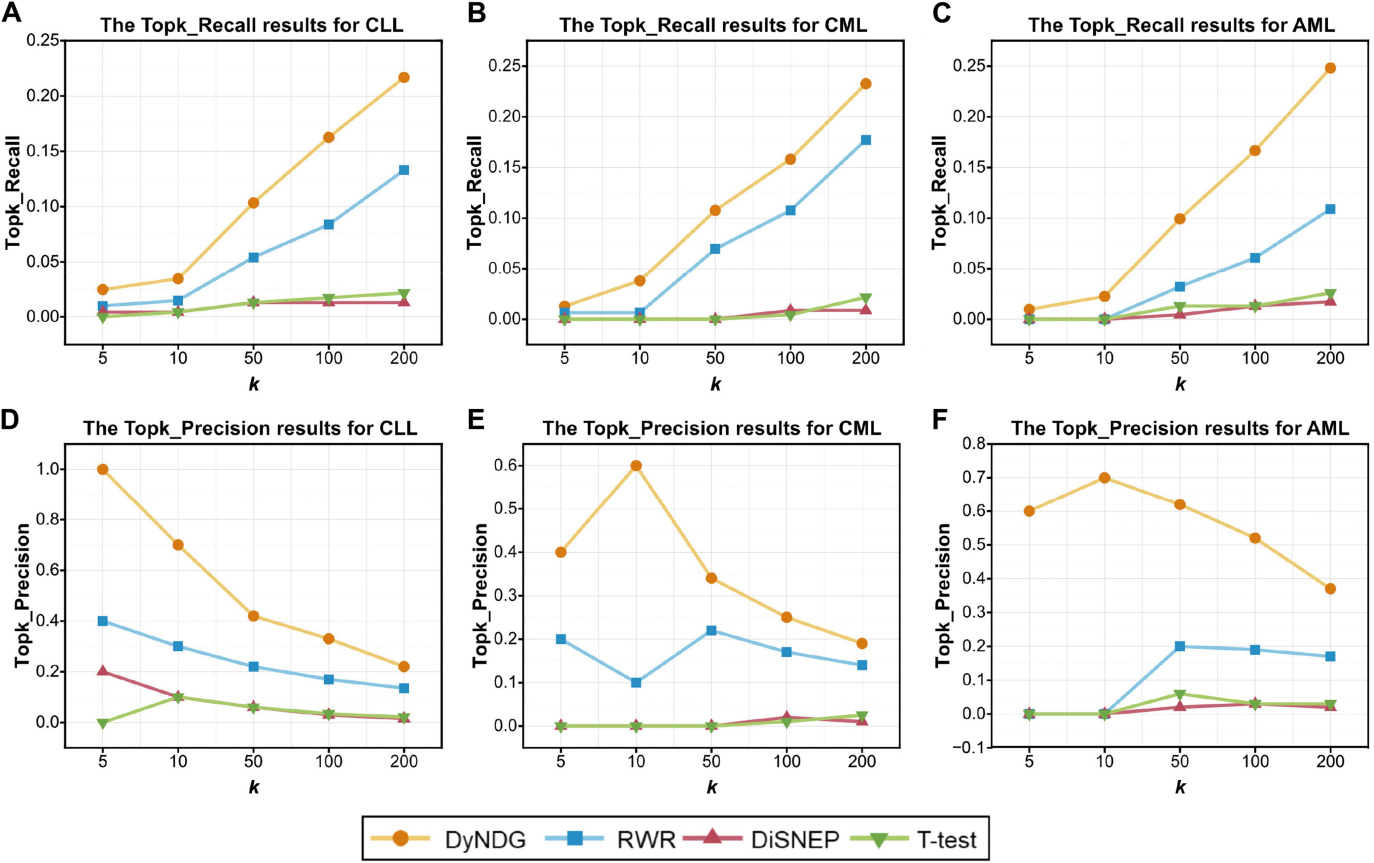
Performance comparison of DyNDG, RWR, T-test, and DiSNEP on WG using Topk_Recall and Topk_Precision **A**.–**C**. The Topk_Recall results for CLL (A), CML (B), and AML (C). **D**.–**F**. The Topk_Precision results for CLL (D), CML (E), and AML (F). When *k* = 5, 10, 50, 100, and 200, DyNDG consistently exhibits higher Topk_Recall and Topk_Precision values than other methods. RWR, Random Walk with Restart; DiSNEP, Disease-Specific Network Enhancement Prioritization; WG, Whole Genomic Control Set; CLL, chronic lymphocytic leukemia; CML, chronic myeloid leukemia; ALL, acute myeloid leukemia.

**Figure 4:**
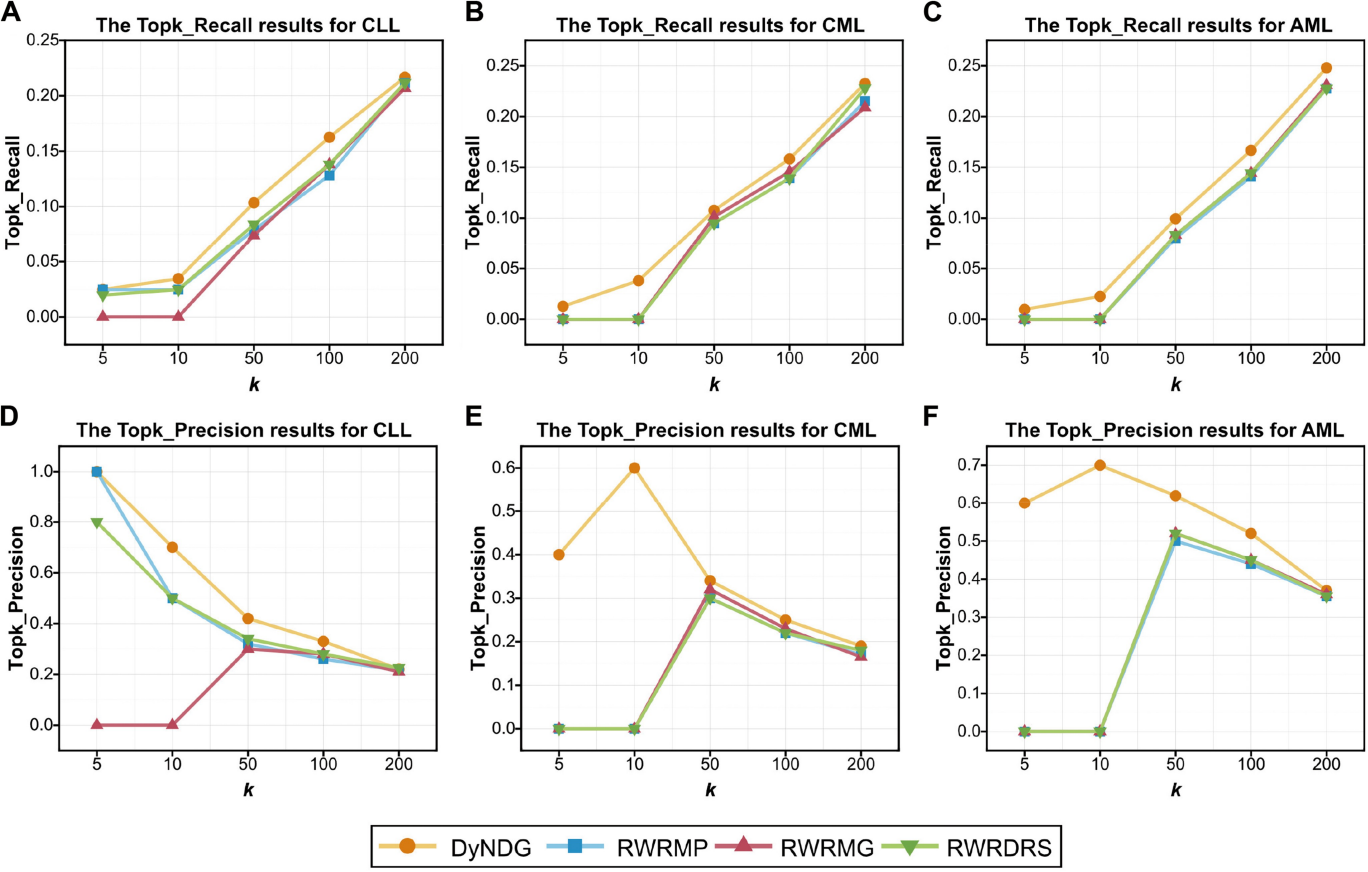
Performance comparison of DyNDG, RWRDRS, RWRMG, and RWRMP on WG using Topk_Recall and Topk_Precision **A**.–**C**. The Topk_Recall results for CLL (A), CML (B), and AML (C). **D**.–**F**. The Topk_Precision results for CLL (D), CML (E), and AML (F). When *k* = 5, 10, 50, 100, and 200, DyNDG consistently outperforms other methods in terms of Topk_Recall and Topk_Precision. RWRDRS, Random Walk with Restart integrating the Discounted Rating System; RWRMG, Random Walk with Restart on the Merged Graph; RWRMP, Random Walk with Restart on Multiplex Graphs.

In identifying leukemia-related genes, our DyNDG method significantly outperforms the single-network-based method RWR [19] based on the static network upon Topk_Recall and Topk_Precision metrics, because of incorporating temporal dynamic network information of disease progression, while RWR demonstrates better performance than the classical T-test method (**Figure 3**). Multi-network-based methods have been designed to operate on multiple independent or interconnected networks, enabling effective utilization of more information from these networks. Here, we also compared our DyNDG method with three multi-network-based methods: RWRDRS [47], RWRMG [18], and RWRMP [20]. The comparison results in **Figure 4**A–C, respectively, show that DyNDG outperforms these multi-network-based methods in identifying CLL-related genes, CML-related genes, and AML-related genes, in terms of Topk_Recall performance. It can be observed that increasing the *k* value appropriately leads to a noticeable improvement in Topk_Recall. It is indeed a difficult task to search for disease-related genes in almost full gene set. The recall scores of all other algorithms are also relatively low. Additionally, if the candidate range for search/prediction is limited, the recall of the algorithm will usually improve, including utilizing more known information. The results in Figure 4D–F also demonstrate that DyNDG can more precisely predict CLL-related genes, CML-related genes, and AML-related genes, respectively, in terms of Topk_Precision performance. Figure S1 shows the overlaps of the top 100 genes predicted by different methods for CLL, CML, and AML. Upon analyzing the overlaps of candidate genes predicted by DyNDG and other methods, we observed more than 50% overlap between DyNDG and other methods, with the majority of the overlapping predictions coming from multi-network methods. Additionally, DyNDG recommended some genes that were not identified by other algorithms, which further illustrates the potential of DyNDG to complement and enhance existing predictive approaches. Figures S2–S5 also show that DyNDG achieves the best on three leukemia datasets and different kinds of the control sets upon Topk_Recall and Topk_Precision, re-affirming that DyNDG can obtain more reliable and stable results, independent to the types of control sets.

Moreover, the results of the performance comparison between DyNDG and other methods, using the AUROC and AUPRC metrics, are shown in **Table 2**. DyNDG significantly outperforms all the comparison algorithms in terms of AUROC and AUPRC, demonstrating a notable advancement in identification of leukemia disease genes.

**Table 2:** Performance comparison of DyNDG and other methods using AUROC and AUPRC metrics

Leukemia	Type	Method	AUROC	AUPRC
CLL	Single-network	RWR	0.8080	0.0646
	Multi-network	RWRMP	0.7967	0.1396
		RWRMG	0.8394	0.1078
		RWRDRS	0.7941	0.1433
	Differential gene expression	T-test	0.7116	0.0224
		DiSNEP	0.7177	0.0231
		**DyNDG**	**0.8875**	**0.1753**
CML	Single-network	RWR	0.7881	0.0676
	Multi-network	RWRMP	0.8487	0.0910
		RWRMG	0.8308	0.0920
		RWRDRS	0.8556	0.0940
	Differential gene expression	T-test	0.5798	0.0162
		DiSNEP	0.5712	0.0154
		**DyNDG**	**0.8983**	**0.1260**
AML	Single-network	RWR	0.7493	0.0745
	Multi-network	RWRMP	0.8422	0.1887
		RWRMG	0.8445	0.1918
		RWRDRS	0.8433	0.1927
	Differential gene expression	T-test	0.5478	0.0155
		DiSNEP	0.5402	0.0145
		**DyNDG**	**0.8797**	**0.2334**

*Note*: DyNDG, Dynamic Network-based model integrating Differentially Expressed Genes; AUROC, area under the receiver operating characteristic curve; AUPRC, area under the precision–recall curve; RWR, Random Walk with Restart; DiSNEP, Disease-Specific Network Enhancement Prioritization; RWRDRS, Random Walk with Restart integrating the Discounted Rating System; RWRMG, Random Walk with Restart on the Merged Graph; RWRMP, Random Walk with Restart on Multiplex Graphs.

### Effect of parameters and data

We systematically examined the impact of parameters (*i.e.*, δ, μ, and γ) on the performance of the DyNDG model in three types of leukemia. Details of the analysis can be found in section 3 of File S1, and the results are presented in Figures S6–S11. δ and μ regulate the transitions of the background–temporal multilayer network. The results indicate that the model performance remains relatively stable as the parameters δ and μ vary, which suggests that the multilayer network framework can effectively integrate background network knowledge and temporal dynamic network information, ensuring the robustness of the model. A lower restart probability γ corresponds to better model performance, indicating that the background–temporal multilayer network contains rich information related to leukemia disease genes and is crucial for predictive performance. Therefore, we chose δ = 0.5, μ = 0.5, and γ=0.1 as the parameter combination that yields the relatively best model performance.

Additionally, we investigated the influence of different static network data on the performance of DyNDG. Further information about this study can be found in section 4 of File S1. The results in Figure S12 show that DyNDG performs better when using the static PPI network of STRING [42], which provides a broader and more diverse range of PPI information. As shown in Figures S13 and S14, we also studied the differences in the initial probability distribution between different layers of the background–temporal multilayer network. The initial probabilities of the background network comprehensively consider the characteristics of the temporal multilayer networks at various stages. The differences in the initial probability distribution across different stages of disease progression indicate that the T-statistic distributions obtained from differential gene analysis at each stage can effectively represent the characteristics of each stage.

To assess the contribution of different components of the DyNDG model to the predictive power, we performed two different experiments: (1) DyNDG_static, where the random walk process was performed only in the background network; and (2) DyNDG_dynet, where the random walk process was conducted exclusively in the dynamic network (detailed experimental setup can be found in section 5 of File S1). Results (Figure S15) show that the background network plays a crucial role in the model which builds upon universal interaction patterns in biomolecules, providing a comprehensive background and a solid foundation for our model. Simultaneously, the introduction of the dynamic network enhances the predictive capability of the model, particularly in simulating dynamic changes in biological networks at different leukemia stages and capturing potential gene associations. This suggests that in the prediction of leukemia-related genes, the collaboration of static and dynamic networks contributes to the overall improvement in predictive performance of the model.

### Comprehensive analysis of candidate disease genes for AML, CLL, and CML

In this study, we further conducted case studies and comprehensive analysis for specific leukemia (*i.e.*, AML, CLL, and CML). Specifically, we applied DyNDG to each of the three leukemias and obtained a ranked list of genes for each of them. Then, we individually filtered each of the three gene lists, by removing the genes reported in MalaCards [44] that are associated with the respective types of leukemia. Additionally, we excluded the human housekeeping genes collected from the Housekeeping and Reference Transcript Atlas (HRT Atlas) database [50]. As a result, we obtained three lists of candidate genes associated with their respective types of leukemia.

#### Integrative analysis of functional annotations and clinical survival outcomes in AML

AML is the most common and lethal adult acute leukemia, characterized by its aggressive nature, poor prognosis, and high susceptibility to relapse after treatment [51]. Through the application of DyNDG, we identified AML-related genes that have the potential to drive AML progression and contribute to poor prognosis in patients.

**Figure 5:**
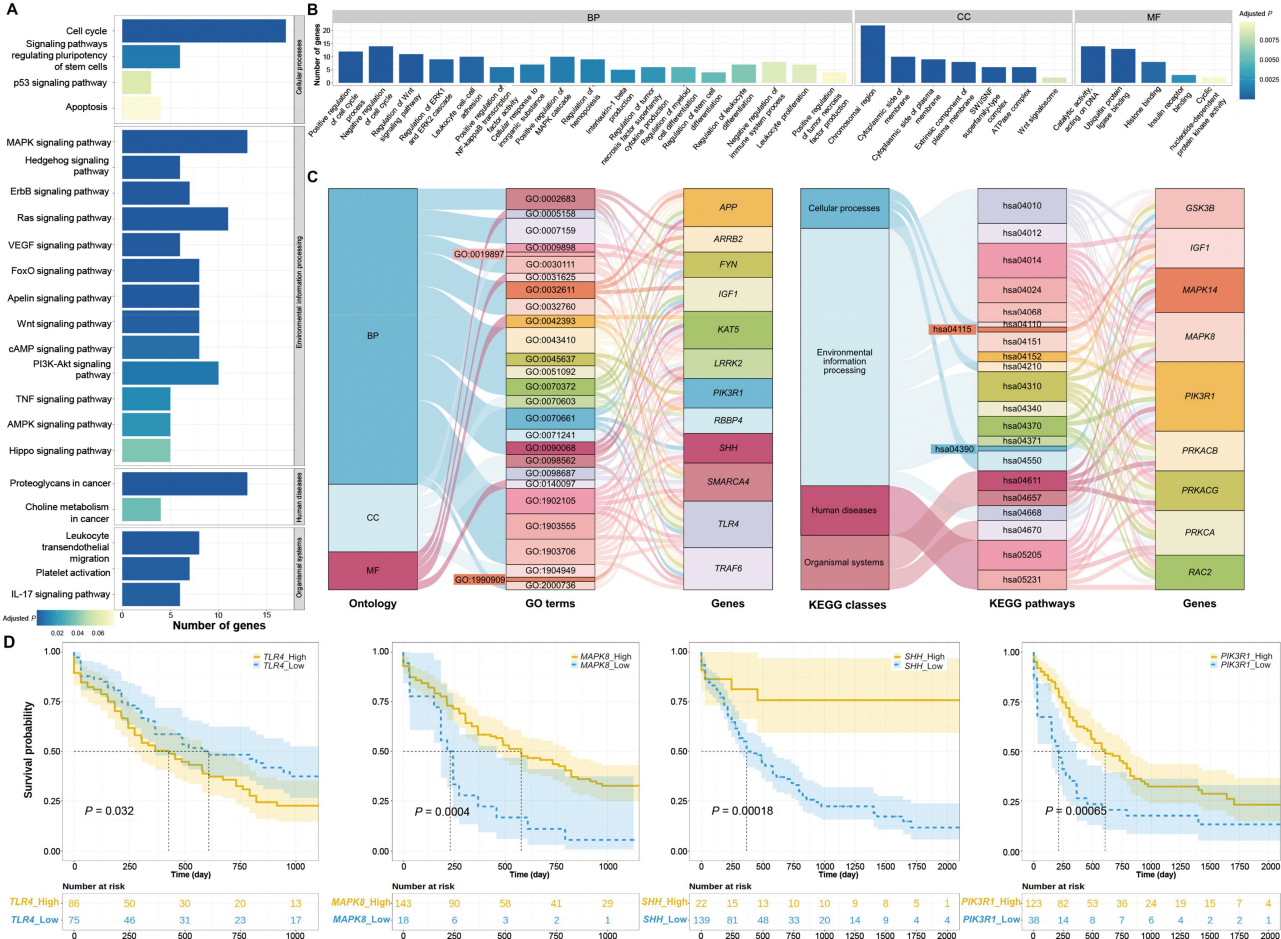
DyNDG predicts AML-related genes closely associated with clinical survival **A**. The bar chart showcases the results of KEGG pathway enrichment analysis, highlighting the most relevant KEGG pathways associated with AML. **B**. The bar chart illustrates the outcomes of GO enrichment analysis, highlighting the most relevant GO terms associated with AML. **C**. The Sankey diagrams show the associations between GO terms and multi-pathway enriched genes, as well as the associations between KEGG pathways and multi-pathway enriched genes. **D**. Kaplan–Meier overall survival curves of AML patients grouped by the averaged expression of *TLR4*, *MAPK8*, *SHH*, and *PIK3R1*, respectively (with the median value as the threshold). *P* values were calculated by the log-rank test. GO, Gene Ontology; KEGG, Kyoto Encyclopedia of Genes and Genomes; BP, biological process; CC, cellular component; MF, molecular function.

Functional enrichment analysis conducted on the top 1% ranked genes in the AML-related candidate gene list revealed the KEGG pathways and GO terms most associated with AML, as shown in **Figure 5**A and B, respectively. The most relevant KEGG pathways consist of cancer-related pathways (*e.g.*, cell cycle and MAPK signaling pathways), signaling pathways regulating pluripotency of stem cells, and leukemia-related pathways (*e.g.*, leukocyte transendothelial migration and platelet activation). The GO enrichment analysis shows many AML-related biological processes (*e.g.*, leukocyte cell–cell adhesion, leukocyte proliferation, and cellular response to inorganic substance), molecular functions (*e.g.*, catalytic activity, acting on DNA and cyclic nucleotide-dependent protein kinase activity), and cellular components (*e.g.*, chromosomal region). To further investigate the candidate genes enriched in these AML-related pathways, we obtained a set of multi-pathway enrichment genes as shown in Figure 5C (corresponding relationships between GO term IDs and descriptions, as well as between KEGG pathway IDs and descriptions, can be found in Table S4).

By conducting survival analysis, we observed significant differences in overall survival among patient groups with different expression levels of many multi-pathway-enriched genes (*e.g.*, *TLR4*, *MAPK8*, *SHH*, and *PIK3R1*) (Figure 5D). *TLR4* is expressed by AML cells, several bone marrow stromal cells, and non-leukemic cells involved in inflammation. *TLR4* in leukemia cells is of significant importance for the growth and development of leukemia cells in human AML, and targeting *TLR4* may have direct and indirect effects on leukemogenesis. A study has indicated that high expression of *TLR4* is associated with a decrease in survival rates after intensified anti-leukemia treatment [52]. This suggests that high expression of *TLR4* may be related to lower survival rates in AML patients, which is consistent with our survival analysis results of *TLR4* shown in Figure 5D. *MAPK8*, also known as *JNK1*, is a member of the MAPK family. The aberrant activation of the MAPK signaling pathway is closely associated with the proliferation, survival, drug resistance, and metastatic capacity of AML cells [53]. Therefore, as a member involved in regulating the MAPK signaling pathway, *MAPK8* may play an important role in key biological processes regulating AML. Studies have shown that in AML, certain anticancer drugs may exert their anticancer effects by activating *MAPK8* to inhibit cell proliferation in AML cell lines [54]. In the clinical survival analysis presented in Figure 5D, it was observed that low expression of *MAPK8* may be associated with lower survival rates. This could be attributed to the aberrant activation or impaired function of the MAPK signaling pathway resulting from the lower expression of *MAPK8*, ultimately leading to reduced survival rates in AML patients. These findings provide important clues for further investigating the role of *MAPK8* in the development and treatment of AML. *SHH* has been implicated in the maintenance of cancer stem cells (CSCs), playing a critical role in the development of drug resistance and disease relapse in AML. Research findings suggest that *SHH* could serve as a prognostic marker for CSCs in AML, providing valuable insights into disease outcomes [55]. Furthermore, in the presence of lipopolysaccharide/tumor necrosis factor-α/interferons (LPS/TNF-α/IFNs), *SHH* antagonists have been utilized for the treatment of AML patients [56]. Previous evidence suggests that *SHH* may be associated with the occurrence, progression, and treatment of AML. As shown in Figure 5D, a significant difference in survival probability was observed between patient groups with different levels of *SHH* expression, providing clinical evidence for the pivotal role of *SHH* in the development of AML. *PIK3R1* is the gene encoding the p85α subunit of the PI3K regulatory subunit, playing a critical regulatory role in the PI3K/Akt signaling pathway. Previous studies have indicated that *PIK3R1* is an actionable gene in AML [57]. The PI3K/Akt signaling pathway regulated by *PIK3R1* is involved in various cellular processes such as cell survival, proliferation, and metabolism in normal cells. Its aberrant activation is associated with the development and progression of multiple tumor types, including AML. In 50%–80% of AML patients, constitutive activation of the PI3K/Akt pathway has been detected, which is associated with reduced overall survival [58]. Therefore, the abnormal expression of *PIK3R1*, which regulates the PI3K/Akt pathway, may be implicated in the occurrence and progression of AML. Figure 5D illustrates the relationship between *PIK3R1* expression and the survival rate of AML. The identification of these genes provides valuable insights into the potential molecular mechanisms, pathogenesis, and key factors influencing the prognosis of AML. It offers opportunities to identify new therapeutic targets and has the potential to improve prognosis assessment and develop personalized treatment strategies.

#### Integrative analysis of functional annotations and networks in CLL

CLL is one of the most prevalent types of leukemia, and there is currently no definitive cure available. Despite advancements in treatment with novel therapies and targeted drugs, there is a continuing need for new targeted treatments and innovative strategies to improve efficacy and survival rates [59]. By applying DyNDG to CLL, we predicted CLL-related genes that can serve as references for discovering new therapeutic targets and developing personalized treatment strategies.

**Figure 6:**
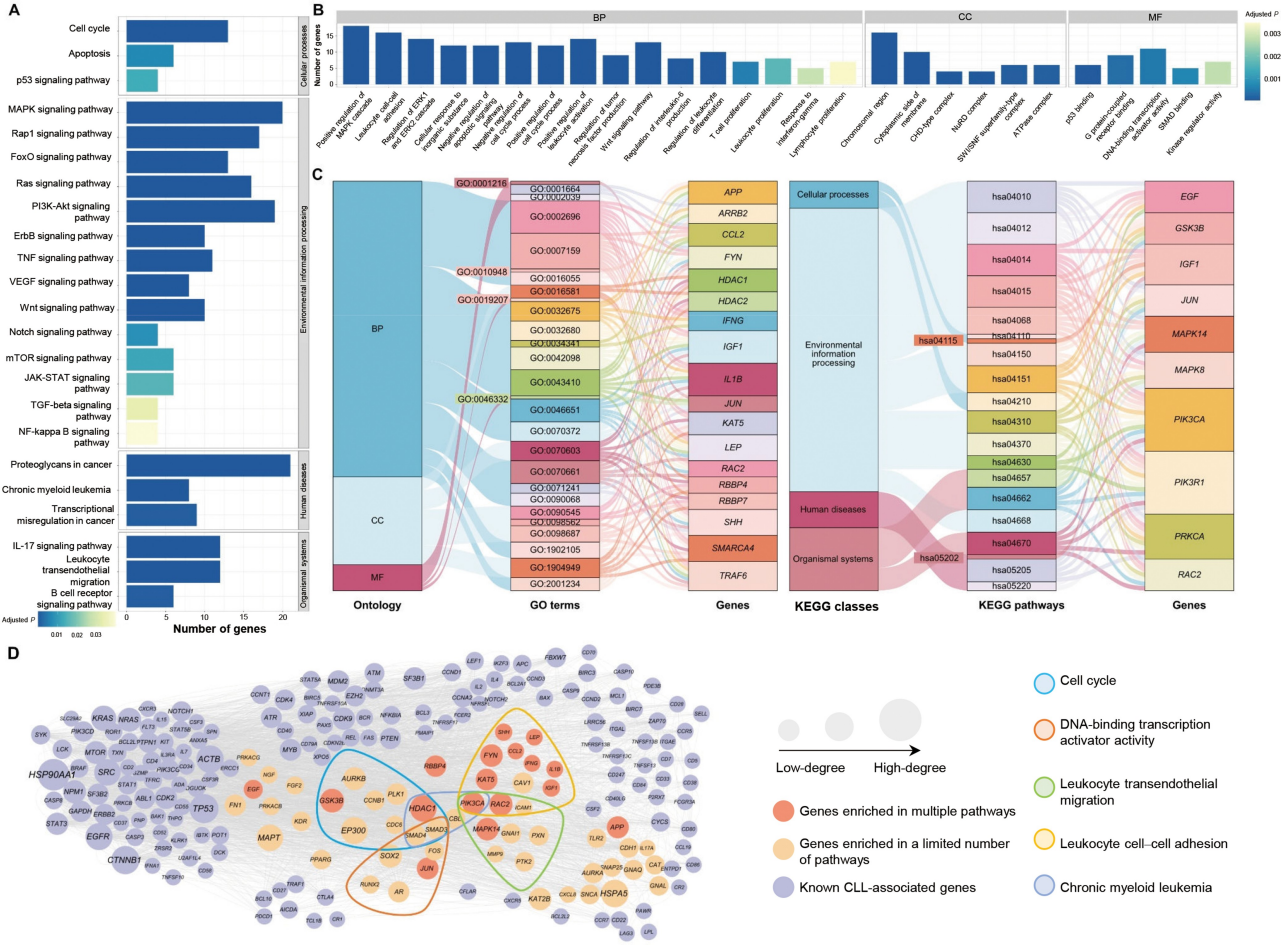
DyNDG predicts CLL-related genes possessing network hub characteristics **A**. The bar chart showcases the results of KEGG pathway enrichment analysis, highlighting the most relevant KEGG pathways associated with CLL. **B**. The bar chart illustrates the outcomes of GO enrichment analysis, highlighting the most relevant GO terms associated with CLL. **C**. The Sankey diagrams show the associations between GO terms and multi-pathway enriched genes, as well as the associations between KEGG pathways and multi-pathway enriched genes. **D**. The network graph reflects the degree centrality of candidate genes enriched in CLL-related pathways and known CLL-related genes.

Functional enrichment analysis conducted on the top 1% candidate genes revealed the KEGG pathways and GO terms most relevant to CLL, as shown in **Figure 6**A and B, respectively. The KEGG pathways most relevant to CLL include cancer-related pathways (such as cell cycle, MAPK signaling pathway, and transcriptional misregulation in cancer), B cell receptor signaling pathway, and pathways associated with chronic leukemias (such as chronic myeloid leukemia and leukocyte transendothelial migration). GO enrichment analysis revealed numerous CLL-related biological processes (such as leukocyte cell–cell adhesion, leukocyte proliferation, lymphocyte proliferation, and regulation of tumor necrosis factor production), molecular functions (such as kinase regulatory activity, SMAD binding, and G protein-coupled receptor binding), and cellular components (such as chromosomal regions). Then, we obtained a set of multi-pathway enriched genes, which are enriched in multiple GO terms and KEGG pathways, through Sankey diagrams as shown in Figure 6C (enriched pathways are identified by their respective GO term IDs and KEGG pathway IDs, which can be found in Table S4, along with their descriptions).

To explore the criticality of candidate genes enriched in CLL-related pathways in the PPI network, we conducted network analysis using Cytoscape [60]. Based on human static PPI network sourced from the STRING database [42], we analyzed the node degree centrality of candidate genes enriched in CLL-related pathways and CLL-related genes reported in MalaCards [44]. Subsequently, we generated a network diagram as shown in Figure 6D. It is well-known that genes with high-degree centrality play a crucial role in regulating and influencing information transmission, network stability, functional integration, and disease relevance within the PPI network. In the static PPI network, candidate genes enriched in CLL-related pathways exhibit degrees similar to known CLL-related genes. This suggests that the candidate genes may have similar network influence as the known CLL-related genes and could play a crucial role in the progression of CLL. Many of these candidate genes even surpass certain known CLL-related genes in terms of degree centrality, indicating that they may have a greater importance in regulating the network than some of the known key genes. *HDAC1*, *PIK3CA*, and *RAC2* are genes that are enriched in multiple pathways and exhibit high-degree centrality in the PPI network. Lastly, we conducted literature research on *HDAC1*, *PIK3CA*, and *RAC2*. *HDAC1* is one of the components of the histone deacetylase complex, and elevated *HDAC* enzyme activity has been found to be associated with the development of leukemia and other cancers. Significantly elevated levels of *HDAC1* expression have been observed in CLL [61], and studies have indicated that *HDAC1* can act as a transcriptional activator in CLL, promoting CLL cell survival and progression [62]. *PIK3CA* is a component of the PI3K pathway and has long been described as an oncogene. Inhibition of PI3K subtype associated with *PIK3CA* has been shown to be potentially valuable in CLL [63]. *RAC2* is a member of the RAC subfamily of Rho GTPases and is closely associated with oncogenic signaling. Existing studies have found that *RAC2* can activate two known PLCγ2 mutations in CLL, suggesting that *RAC2* may play a significant role in the survival and proliferation of CLL cells carrying these mutations [64]. Research has also indicated that upregulation of *RAC2* expression may be associated with resistance to ibrutinib in CLL [65]. In conclusion, *HDAC1*, *PIK3CA*, and *RAC2* may have significant biological functions and clinical implications in CLL. They could potentially serve as potential therapeutic targets or biomarkers for predicting CLL progression and treatment response.

#### Integrative analysis of functional annotations and differential expression in CML

**Figure 7:**
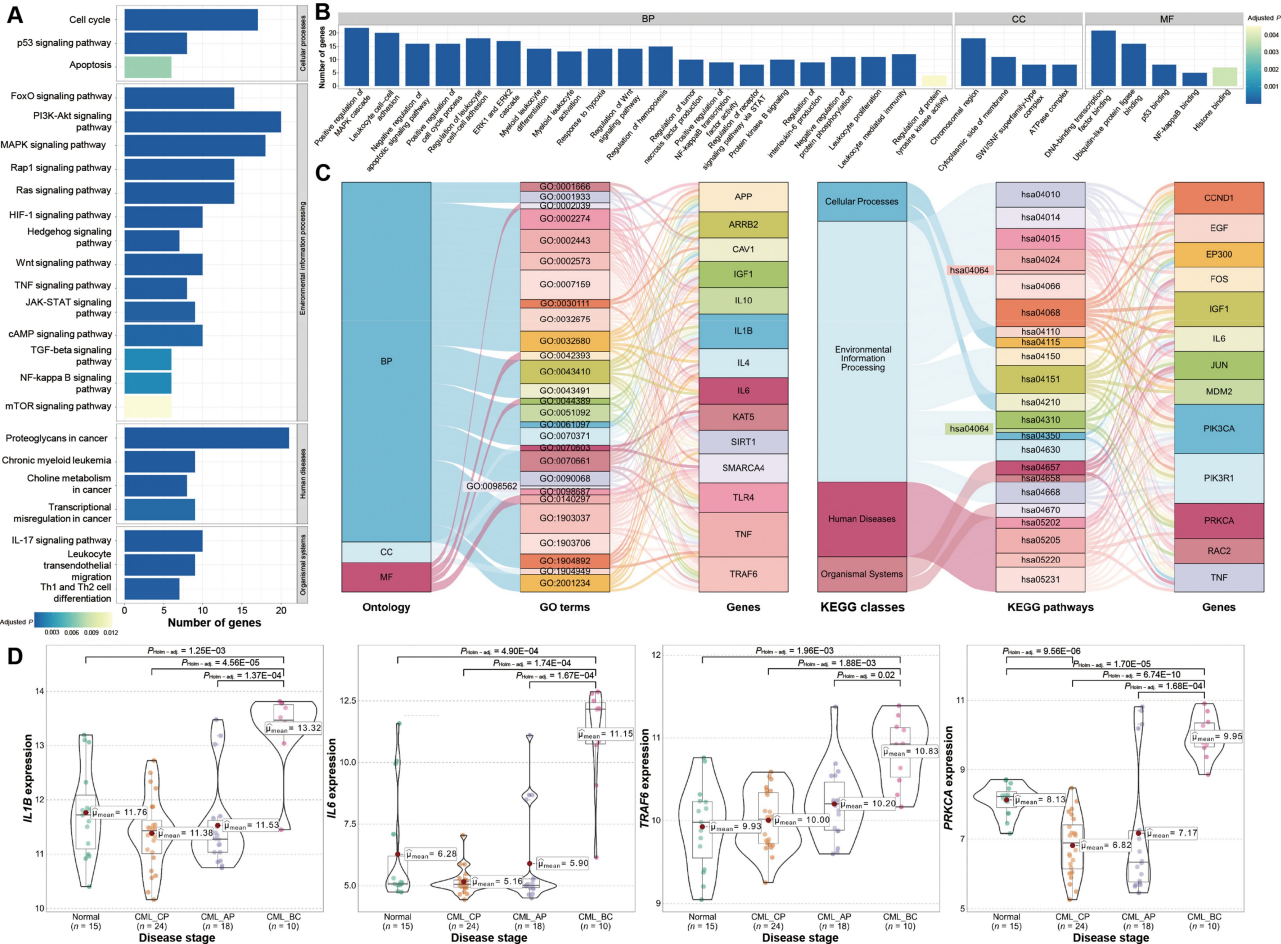
DyNDG predicts CML-related genes closely associated with disease progression **A**. The bar chart showcases the results of KEGG pathway enrichment analysis, highlighting the most relevant KEGG pathways associated with CML. **B**. The bar chart illustrates the outcomes of GO enrichment analysis, highlighting the most relevant GO terms associated with CML. **C**. The Sankey diagrams show the associations between GO terms and multi-pathway enriched genes, as well as the associations between KEGG pathways and multi-pathway enriched genes. **D**. The violin plots display the significant differences between CML disease progression stages for *IL1B*, *IL6*, *TRAF6*, and *PRKCA*, respectively. CP, chronic phase; AP, accelerated phase; BP, blast phase.

CML is a chronic leukemia caused by the fusion of the *BCR* and *ABL* genes. Inhibiting the activity of the BCR–ABL fusion protein with tyrosine kinase inhibitors (TKIs) can effectively control the proliferation and survival of CML cells, leading to sustained clinical and molecular remission in the majority of patients. However, long-term use of TKIs may result in side effects, and some patients may develop resistance or intolerance to TKI treatment [66]. By applying DyNDG to CML, predicting genes associated with its disease progression may provide valuable insights for studying new targets in CML, addressing the issue of drug resistance, and exploring novel therapeutic approaches for CML. Functional enrichment analysis conducted on the top 1% candidate genes revealed the KEGG pathways and GO terms most related to CML, as shown in **Figure 7**A and B, respectively.

The KEGG pathways most relevant to CML include cancer-related pathways (such as cell cycle, PI3K-Akt signaling pathway, and proteoglycans in cancer), the pathways closely associated with the immunity (such as Th1 and Th2 cell differentiation), and pathways associated with chronic leukemias (such as chronic myeloid leukemia and leukocyte transendothelial migration). GO enrichment analysis revealed numerous CML-related biological processes (such as leukocyte cell–cell adhesion, leukocyte proliferation, myeloid leukocyte differentiation, and leukocyte mediated immunity), molecular functions (such as DNA-binding transcription factor binding and ubiquitin-like protein ligase binding), and cellular components (such as chromosomal regions). In order to identify key genes for further research, we generated Sankey diagrams, as shown in Figure 7C, to obtain genes enriched in multiple GO terms and KEGG pathways (IDs and corresponding descriptions of GO terms and KEGG pathways can be found in Table S4).

Then, by studying the expression level changes of these key genes in different pathological stages of CML, we found significant differences in the expression of many genes across different stages of CML development. Specifically, *IL1B*, *IL6*, *TRAF6*, and *PRKCA* show significantly higher expression levels in the CML_BP stage than in other stages (Figure 7D), indicating their association with CML disease progression and their potential as specific markers for the late-stage of CML. The extensive previous research can provide support for our analysis. IL-1β is an important member of the IL-1 family and serves as a crucial cytokine involved in various inflammatory processes and certain anti-tumor physiological mechanisms. The increase in *IL1B* levels may promote CML resistance to TKIs by enhancing cell viability and facilitating cell migration [67]. Recent studies have shown that the development of CML is accompanied by elevated levels of *IL1B* [68]. IL-6, a pleiotropic cytokine, plays an important role in some processes associated with leukemia such as immune response and hematopoiesis. *IL6* has been regarded as a valuable prognostic marker in CML and may be correlated with the transformation of different phases in CML because *IL6* expression in CML-BP is significantly upregulated compared with that in CML-CP and CML-AP [69]. TRAF6 is a member of the superfamily of TRAF proteins. It is not only overexpressed in many cancer tissues but also closely associated with tumor cell proliferation, migration, and apoptosis. Studies have shown that interfering with or inhibiting the role of *TRAF6* in tumor-related signaling pathways may provide new therapeutic approaches for cancer treatment [70]. In patients with CML undergoing imatinib treatment, downregulation of *TRAF6* leads to higher levels of cellular apoptosis, indicating the significant regulatory role of *TRAF6* in CML cell survival and treatment response [71]. *PRKCA*, which encodes PKCα, is an important subtype of the protein kinase C (PKC) family. Aberrant regulation of different PKC subtypes is associated with the development of many human diseases. In CML cells, PKCα, as a classical PKC isoform, exhibits significantly reduced kinase activity. This indicates that PKCα may be functionally impaired or inactivated in CML, potentially contributing to abnormal proliferation and pathological progression of CML cells [72].

### Genetic dependence analysis in AML, CLL, and CML

**Figure 8:**
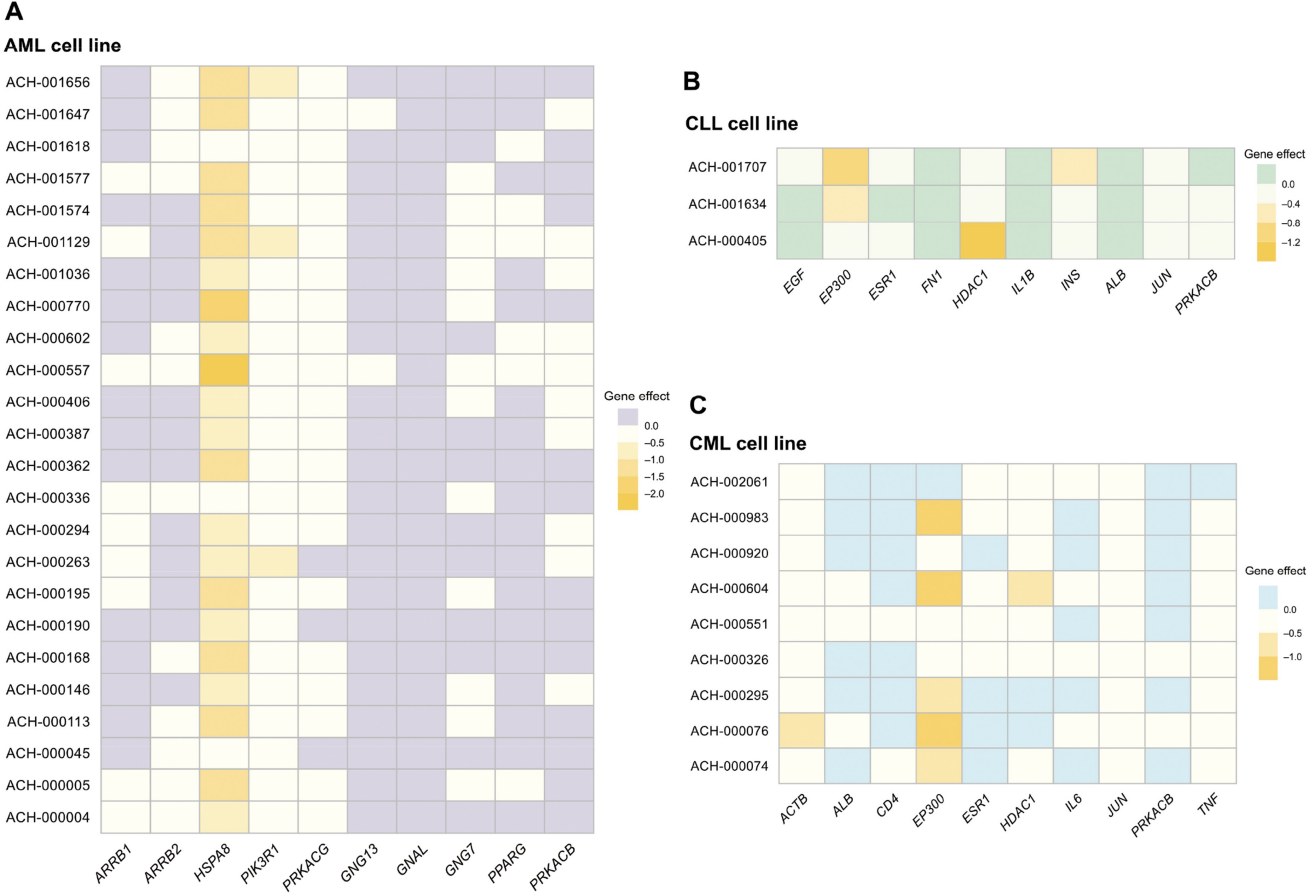
Gene effect profiles of the top 10 predicted candidate genes for three types of leukemia The heatmaps show the “Gene effect” values for the top 10 DyNDG-predicted candidate genes in AML cell lines (**A**), CLL cell lines (**B**), and CML cell lines (**C**).

To verify the effectiveness of our method DyNDG, we utilized the independent DepMap database [73] and investigated the roles of predicted candidate genes in different types of leukemia cell lines. Specifically, we analyzed three types of leukemia cell lines: AML, CLL, and CML, focusing on the top 10 genes in our predicted candidate gene lists. Notably, the “Gene effect” values for most of these genes are below 0, with some even lower than −1 (**Figure 8**A–C), which suggests that the majority of the top 10 predicted genes are likely essential in the corresponding leukemia cell lines, as knocking out or down these genes can affect leukemia cell proliferation. Almost all of the top 10 predicted genes are associated with at least one type of leukemia cell lines, where their knockout or knockdown shows significant effects. Some of these genes have been implicated in the diagnosis and prognosis of leukemia. For example, it has been shown that high expression of *HSPA8* is often associated with poorer survival rates in AML patients [74]; *HDAC1* can activate driver genes in CLL, thereby promoting the survival and progression of CLL [62]; *EP300* is a promising tumor therapeutic target. The mutations of *EP300* frequently occur in various types of hematologic malignancies, including pediatric ALL (both primary and relapsed), aggressive natural killer-cell leukemia (ANKL), and CML-CP, through diverse mechanisms [75]. These findings not only confirm the efficacy of our predictive method but also highlight the potential of these predicted candidate genes as therapeutic targets. Moreover, we examined the “Gene effect” of known disease genes for AML, CLL, and CML in the DepMap database. As shown in Figure S16A–C, most known disease genes display a significant “Gene effect” on leukemia cell proliferation, while a few show no measurable impact in the available cell line datasets. These findings are consistent with the results of our analysis of the predicted candidate genes. This further confirms the potential relevance of our predicted candidate genes to leukemia and validates the effectiveness of our prediction method.

## Conclusion

The occurrence and development of blood diseases, such as leukemia, are closely related to gene mutations and abnormal expression. While existing methods for predicting disease genes have made significant contributions, most of them rely on static networks, limiting the improvement of their predictive ability. Addressing how to integrate dynamic information during disease development is a crucial issue that needs to be solved. Here, we proposed the dynamic network-based model called DyNDG for predicting leukemia-related genes, which consists of three main steps: (1) constructing a time-series dynamic network; (2) building a background–temporal multilayer biological network; and (3) applying a novel network propagation approach on the background–temporal multilayer network to generate a ranked list of leukemia-related genes.

The DyNDG model was evaluated on CLL-related, CML-related, and AML-related datasets. Performance was assessed using four metrics: Topk_Precision, Topk_Recall, AUROC, and AUPRC. Comparing to classical single-network and multi-network methods as well as differential gene expression-based methods, DyNDG shows superior performance in predicting leukemia-related genes.

Finally, we processed the DyNDG-predicted candidate genes for AML, CLL, and CML, respectively, by excluding known leukemia-related genes and housekeeping genes, to generate three candidate gene lists for the three types of leukemia. We systematically analyzed the candidate genes for the three types of leukemia from different perspectives by integrating various analysis methods. The results demonstrate that the predicted candidate genes possess significant research value across various biological contexts. These findings further emphasize DyNDG’s comprehensive predictive capability, multifunctionality, and robustness in accurately identifying disease genes across multiple types of leukemia.

## Code availability

The implementation of DyNDG is available at https://github.com/CSUBioGroup/DyNDG. The code has also been submitted to BioCode at the National Genomics Data Center (NGDC), China National Center for Bioinformation (CNCB) (BioCode: BT007617), which is publicly accessible at https://ngdc.cncb.ac.cn/biocode/tool/BT7617.

## Supplementary Material

qzaf037_Supplementary_Data

## Data Availability

The web server of DyNDG can be freely accessed at https://csuligroup.com/DyNDG.

## CRediT author statement

**Jin A:** Conceptualization, Data curation, Formal analysis, Methodology, Visualization, Writing – original draft. **Ju Xiang:** Formal analysis, Methodology, Writing – original draft. **Xiangmao Meng:** Data curation, Formal analysis. **Yue Sheng:** Supervision. **Hongling Peng:** Supervision. **Min Li:** Formal analysis, Writing – review & editing, Methodology, Supervision. All authors have read and approved the final manuscript.
