# Supplementary material for: DyNDG: Identifying Leukemia-related Genes Based on Time-series Dynamic Network by Integrating Differential Genes
Source: Genomics Proteomics Bioinformatics. 2025 Apr 29;23(2):qzaf037. doi: 10.1093/gpbjnl/qzaf037 (PMC12417087; doi:10.1093/gpbjnl/qzaf037)
Supplement: qzaf037_Supplementary_Data [file qzaf037_supplementary_data.zip › supplementary material captions.docx]

**Supplementary material**

**File S1 Description of the Equation and experimental details**

**Figure S1 The overlaps of the top 100 genes predicted by different comparison methods**

**A.** Upset plot for CLL. **B.** Upset plot for CML. **C.** Upset plot for AML. **D.** Venn diagram for CLL. **E.** Venn diagrams for CML. **F.** Venn diagrams for AML.

**Figure S2 Performance comparison of DyNDG, RWR, T-test and DiSNEP under ALI control set: Topk_Recall and Topk_Precision**

**A.** The Topk_Recall results for CLL. **B.** The Topk_Recall results for CML. **C.** The Topk_Recall results for AML. **D.** The Topk_Precision results for CLL. **E.** The Topk_Precision results for CML. **F.** The Topk_Precision results for AML. When k is 5, 10, 50, 100, and 200, DyNDG consistently outperforms other baseline methods in terms of Topk_Recall and Topk_Precision. ALI, Artificial Linked Interval Control Set.

**Figure S3 Performance comparison of DyNDG, RWR, T-test and DiSNEP under RC control set: Topk_Recall and Topk_Precision**

**A.** The Topk_Recall results for CLL. **B.** The Topk_Recall results for CML. **C.** The Topk_Recall results for AML. **D.** The Topk_Precision results for CLL. **E.** The Topk_Precision results for CML. **F.** The Topk_Precision results for AML. When k is 5, 10, 50, 100, and 200, DyNDG consistently achieves higher Topk_Recall and Topk_Precision values compared to other methods. RC, Randomized Control Set.

**Figure S4 Performance comparison of DyNDG, RWRDRS, RWRMG, and RWRMP under ALI control set: Topk_Recall and Topk_Precision**

**A.** The Topk_Recall results for CLL. **B.** The Topk_Recall results for CML. **C.** The Topk_Recall results for AML. **D.** The Topk_Precision results for CLL. **E.** The Topk_Precision results for CML. **F.** The Topk_Precision results for AML. When k is 5, 10, 50, 100, and 200, the Topk_Recall and Topk_Precision values of DyNDG are higher than other methods.

**Figure S5 Performance comparison of DyNDG, RWRDRS, RWRMG, and RWRMP under RC control set: Topk_Recall and Topk_Precision**

**A.** The Topk_Recall results for CLL. **B.** The Topk_Recall results for CML. **C.** The Topk_Recall results for AML. **D.** The Topk_Precision results for CLL. **E.** The Topk_Precision results for CML. **F.** The Topk_Precision results for AML. When k is 5, 10, 50, 100, and 200, DyNDG consistently demonstrates superior performance compared to other methods in terms of both Topk_Recall and Topk_Precision.

**Figure S6** **Predictive performance of DyNDG for CML-related genes prediction with different parameter settings under AUROC**

The AUROC metric of the DyNDG model was examined to assess its sensitivity to the parameters $\delta$, $\mu$, and $\gamma$ in the context of CML using the WG control set. AUROC, area under the receiver operating characteristic curve.

**Figure S7 Predictive performance of DyNDG for CML-related genes prediction with different parameter settings under AUPRC**

The AUPRC metric of the DyNDG model was analyzed to evaluate how it varied with different values of parameters $\delta$, $\mu$, and $\gamma$ in the context of CML using the WG control set. AUPRC, area under the precision–recall curve.

**Figure S8 Predictive performance of DyNDG for CLL-related genes prediction with different parameter settings under AUROC**

The AUROC metric of the DyNDG model was investigated to evaluate its dependence on the parameters $\delta$, $\mu$, and $\gamma$ in CLL using the WG control set.

**Figure S9 Predictive performance of DyNDG for CLL-related genes prediction with different parameter settings under AUPRC**

The AUPRC metric of the DyNDG model was examined to explore how it was influenced by different parameters $\delta$, $\mu$, and $\gamma$ in CLL using the WG control set.

**Figure S10 Predictive performance of DyNDG for AML-related genes prediction with different parameter settings under AUROC**

The AUROC metric of the DyNDG model was investigated to assess its sensitivity to different parameters $\delta$, $\mu$, and $\gamma$ in AML using the WG control set.

**Figure S11 Predictive performance of DyNDG for AML-related genes prediction with different parameter settings under AUPRC**

The AUPRC metric of the DyNDG model was analyzed to explore how it varied with different parameters $\delta$, $\mu$, and $\gamma$ in AML using the WG control set.

**Figure S12 Comparative analysis of the predictive performance of DyNDG using different static PPI networks**

The impact of different static PPI networks (HumanNet and STRING) on the predictive performance of DyNDG for three leukemia. **A.** The AUROC values for CLL. **B.** The AUROC values for CML. **C.** The AUROC values for AML. **D.** The AUPRC values for CLL. **E.** The AUPRC values for CML. **F.** The AUPRC values for AML. The findings demonstrate that the model achieves superior performance when utilizing the STRING static PPI network.

**Figure S13 The distribution of absolute T-statistics in each network layer of the background temporal multilayer network**

**A.** The distribution of absolute T-statistics in each layer for AML. **B.** The distribution of absolute T-statistics in each layer for CML. **C.** The distribution of absolute T-statistics in each layer for CLL. **D.** The absolute values of T-statistics for different network layers from the gene dimension for AML. **E.** The absolute values of T-statistics for different network layers from the gene dimension for CML. **F.** The absolute values of T-statistics for different network layers from the gene dimension for CLL.

**Figure S14 The correlation of T-statistics distribution between different network layers**

**A.** The correlation for AML. **B.** The correlation for CML. **C.** The correlation for CLL.

**Figure S15 The results of ablation study**

**A.** The AUROC values for CLL. **B.** The AUROC values for CML. **C.** The AUROC values for AML. **D.** The AUPRC values for CLL. **E.** The AUPRC values for CML. **F.** The AUPRC values for AML.

**Figure S16 The “Gene effect” values of cancer gene sets for three types of leukemia**

The heat map shows the “Gene effect” values in corresponding cell lines for the cancer gene sets for three types of leukemia. **A.** Cell lines of AML. **B.** Cell lines of CLL. **C.** Cell lines of CML.

**Table S1 The details of the static PPI networks**

**Table S2 Data composition of the constructed background–temporal multilayer network**

**Table S3 Algorithms for disease–gene prediction**

**Table S4 Enriched pathways and enriched GO terms for predicted top 1% candidate genes for CLL, CML, and AML.**
