# Supplementary figures and images for: DyNDG: Identifying Leukemia-related Genes Based on Time-series Dynamic Network by Integrating Differential Genes
Source: Genomics Proteomics Bioinformatics. 2025 Apr 29;23(2):qzaf037. doi: 10.1093/gpbjnl/qzaf037 (PMC12417087; doi:10.1093/gpbjnl/qzaf037)

## Slide 1
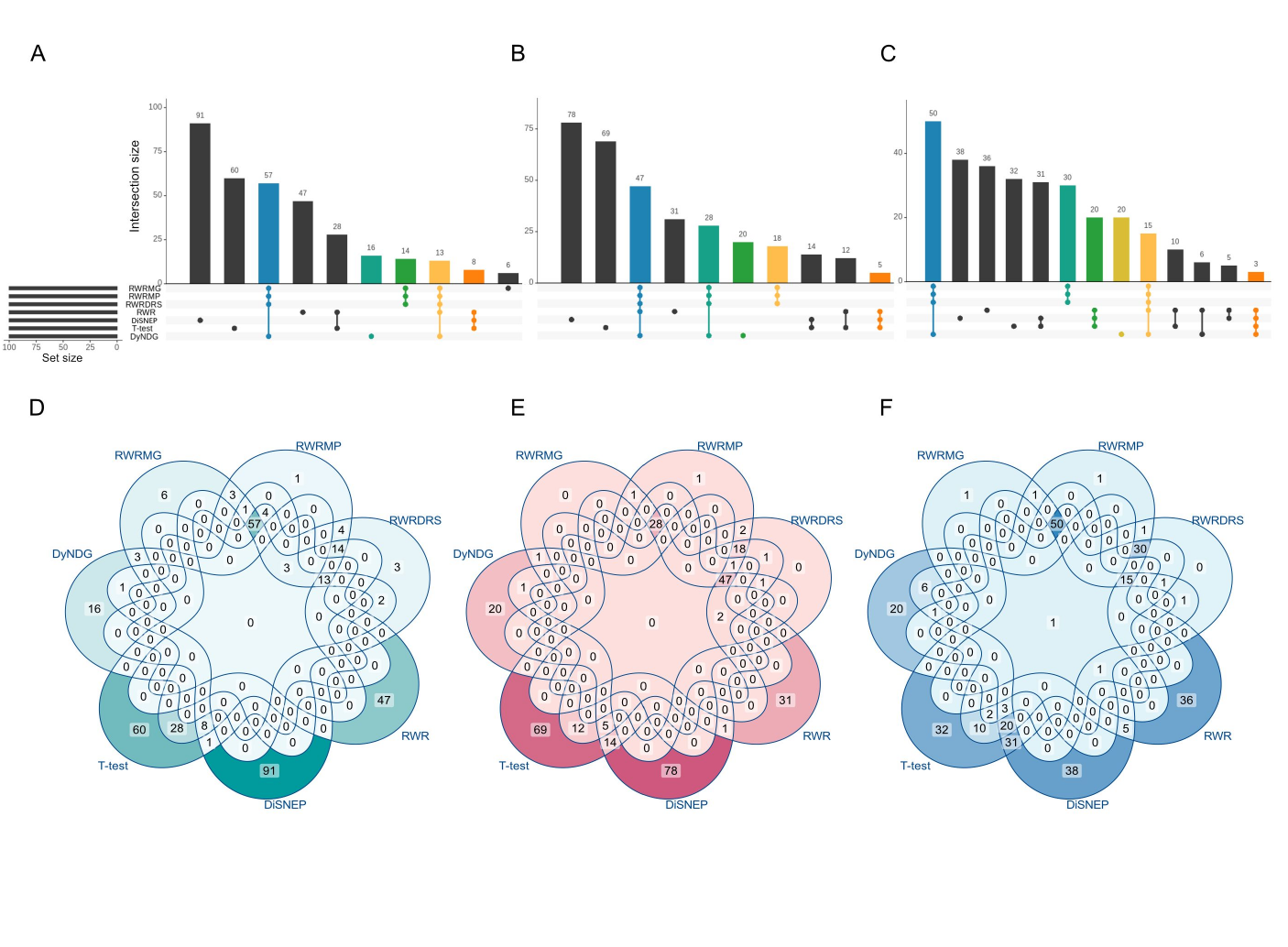

Supplement: qzaf037_Supplementary_Data [file qzaf037_supplementary_data.zip › Figure S1.pptx]

## Slide 1
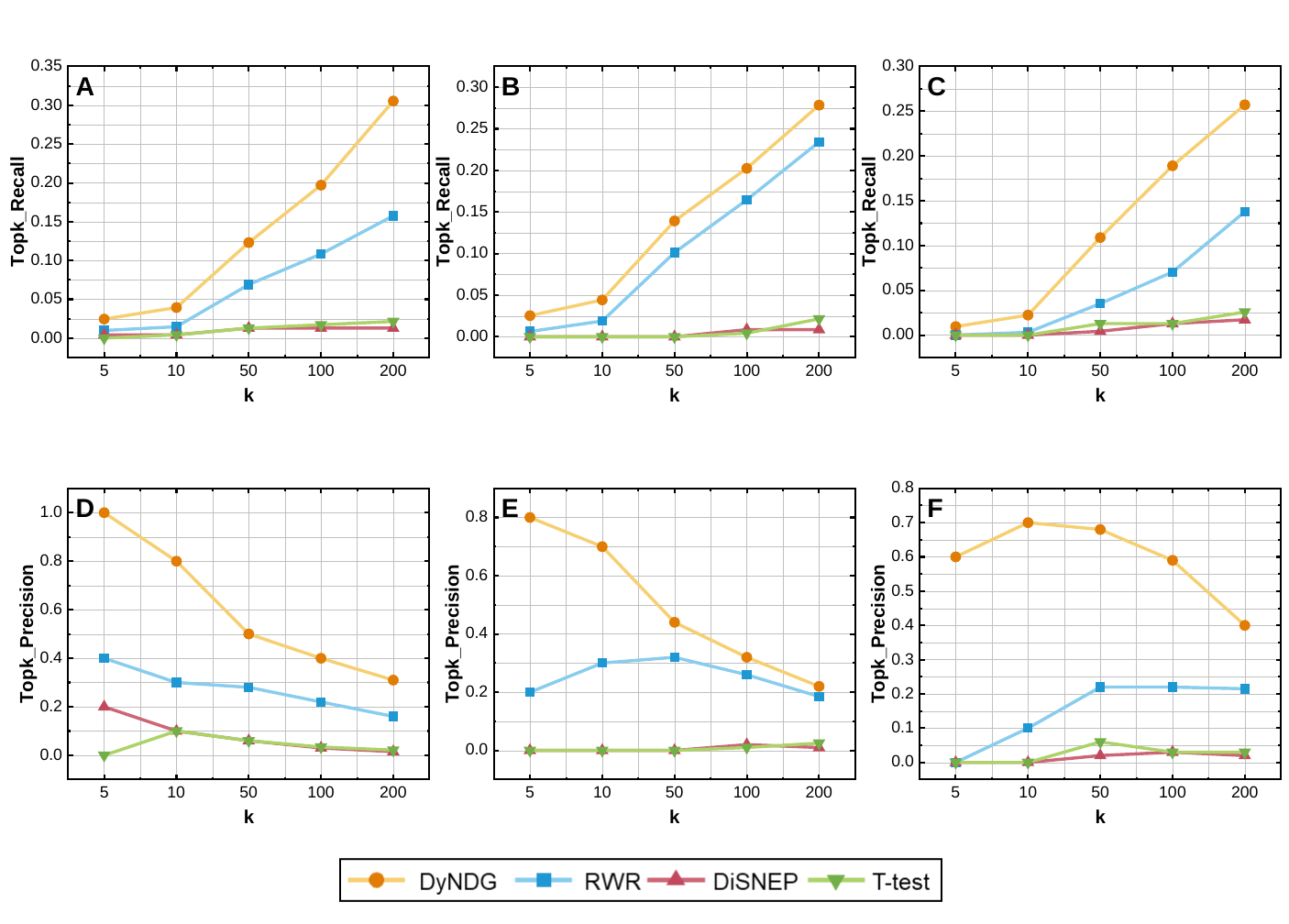

Supplement: qzaf037_Supplementary_Data [file qzaf037_supplementary_data.zip › Figure S2.pptx]

## Slide 1
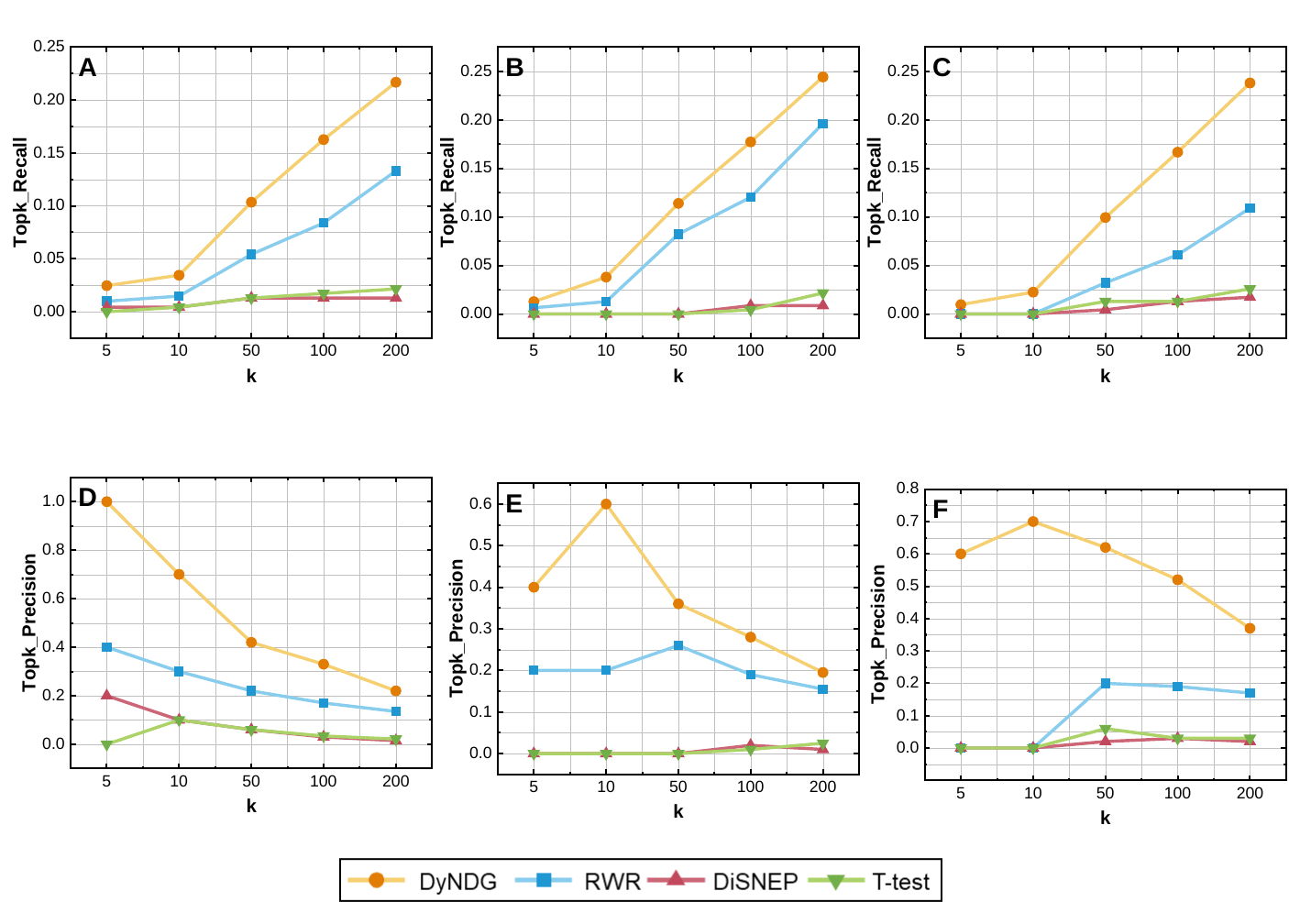

Supplement: qzaf037_Supplementary_Data [file qzaf037_supplementary_data.zip › Figure S3.pptx]

## Slide 1
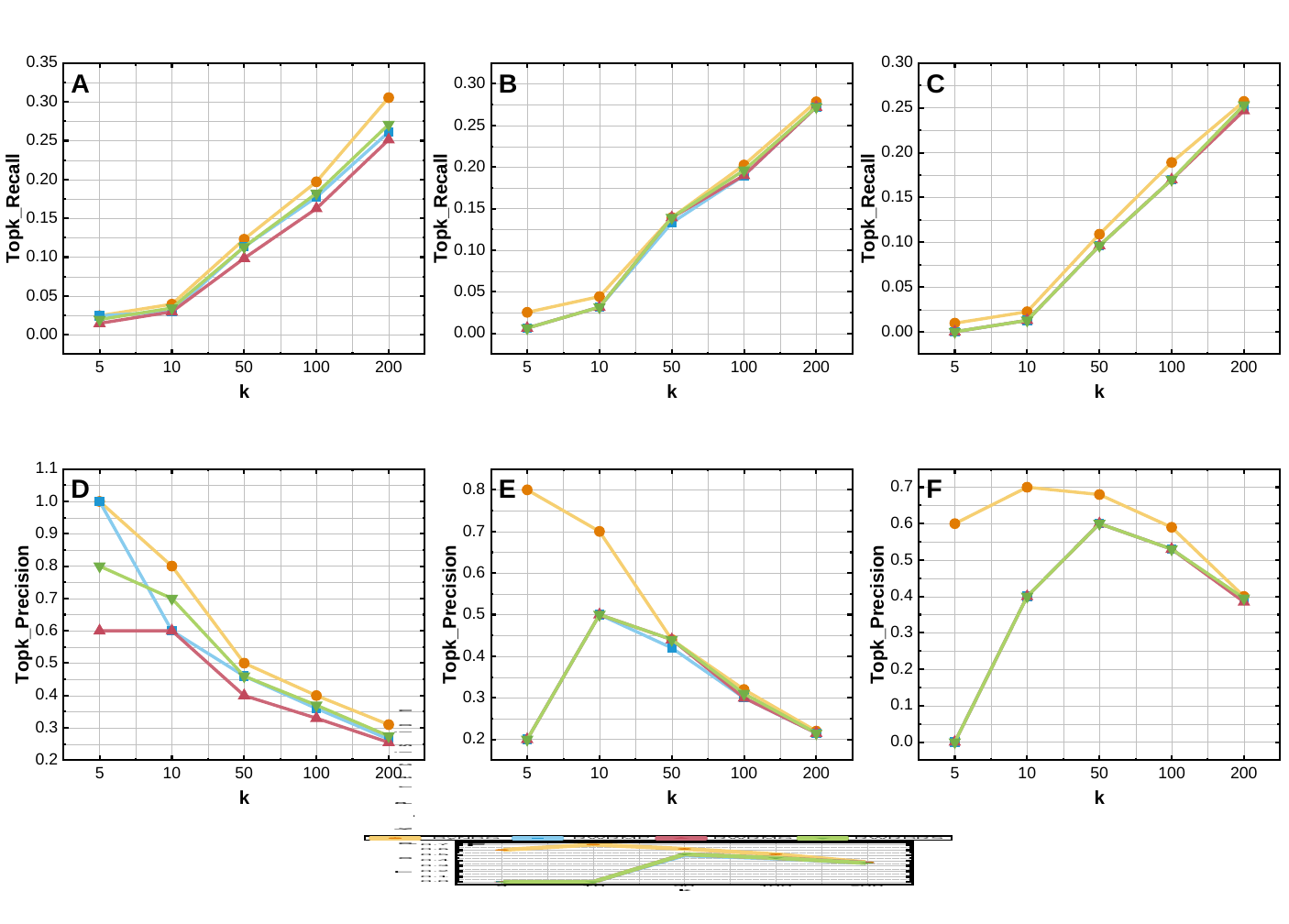

Supplement: qzaf037_Supplementary_Data [file qzaf037_supplementary_data.zip › Figure S4.pptx]

## Slide 1
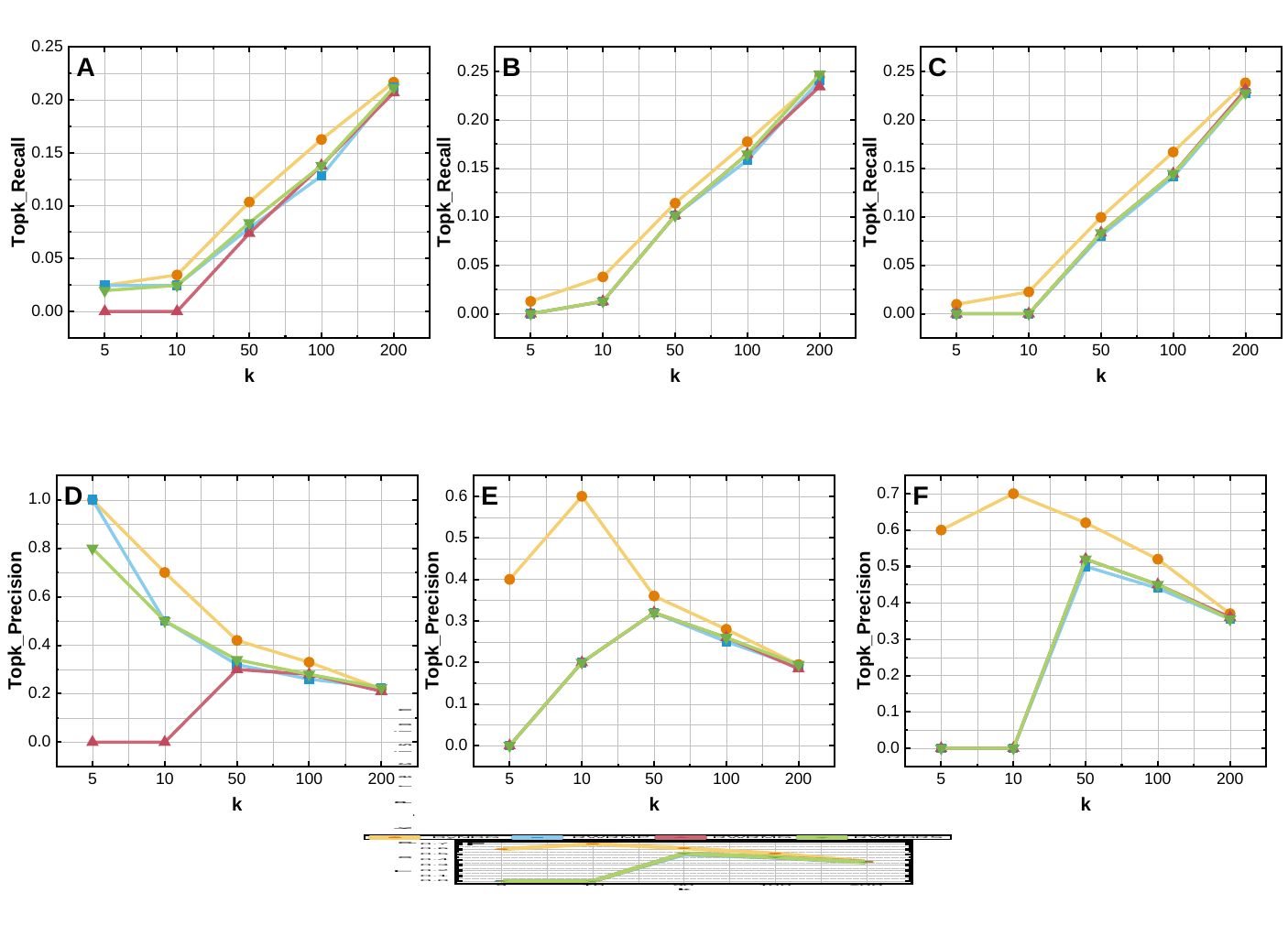

Supplement: qzaf037_Supplementary_Data [file qzaf037_supplementary_data.zip › Figure S5.pptx]

## Slide 1
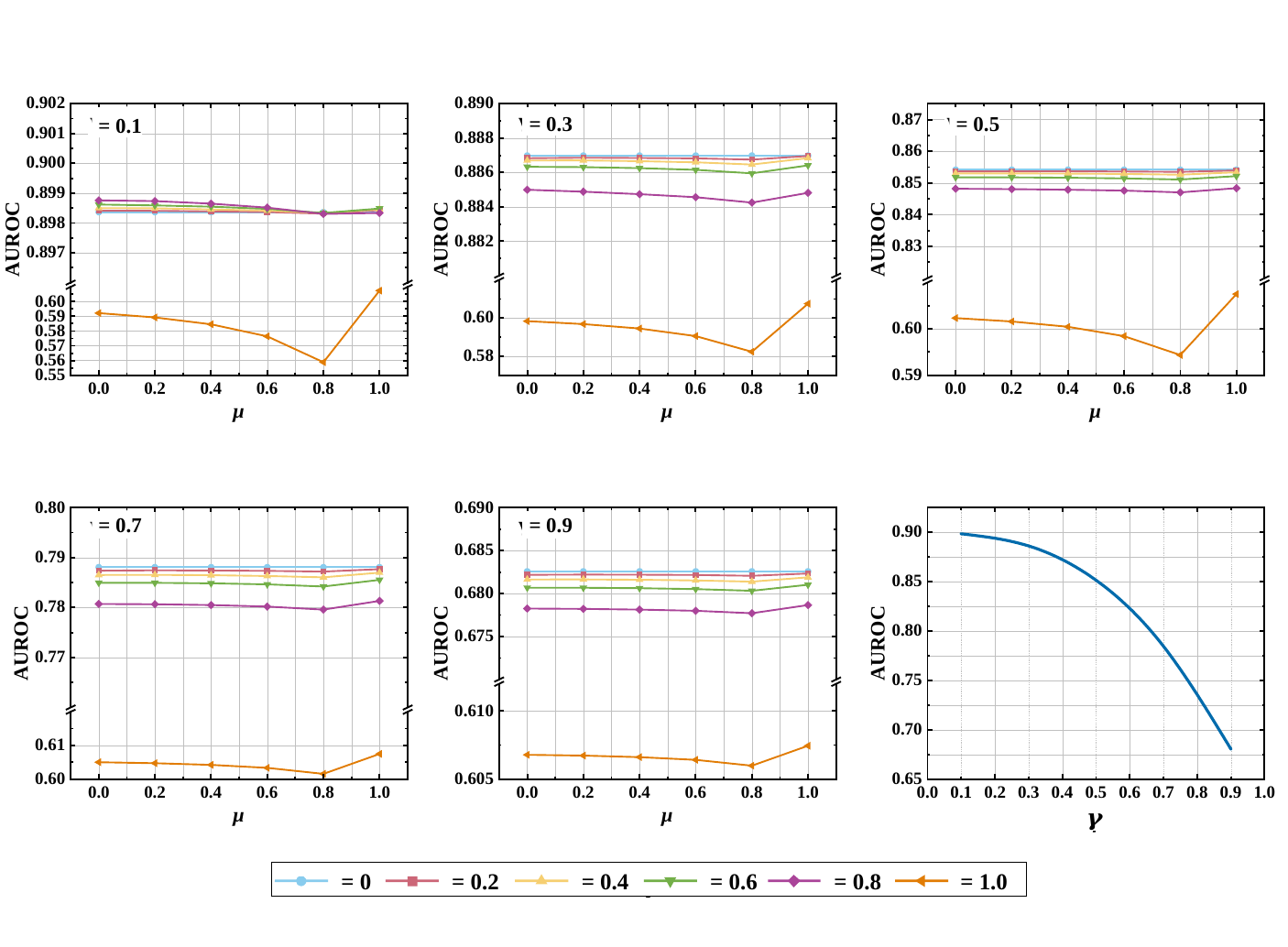

µ

Supplement: qzaf037_Supplementary_Data [file qzaf037_supplementary_data.zip › Figure S6.pptx]

## Slide 1
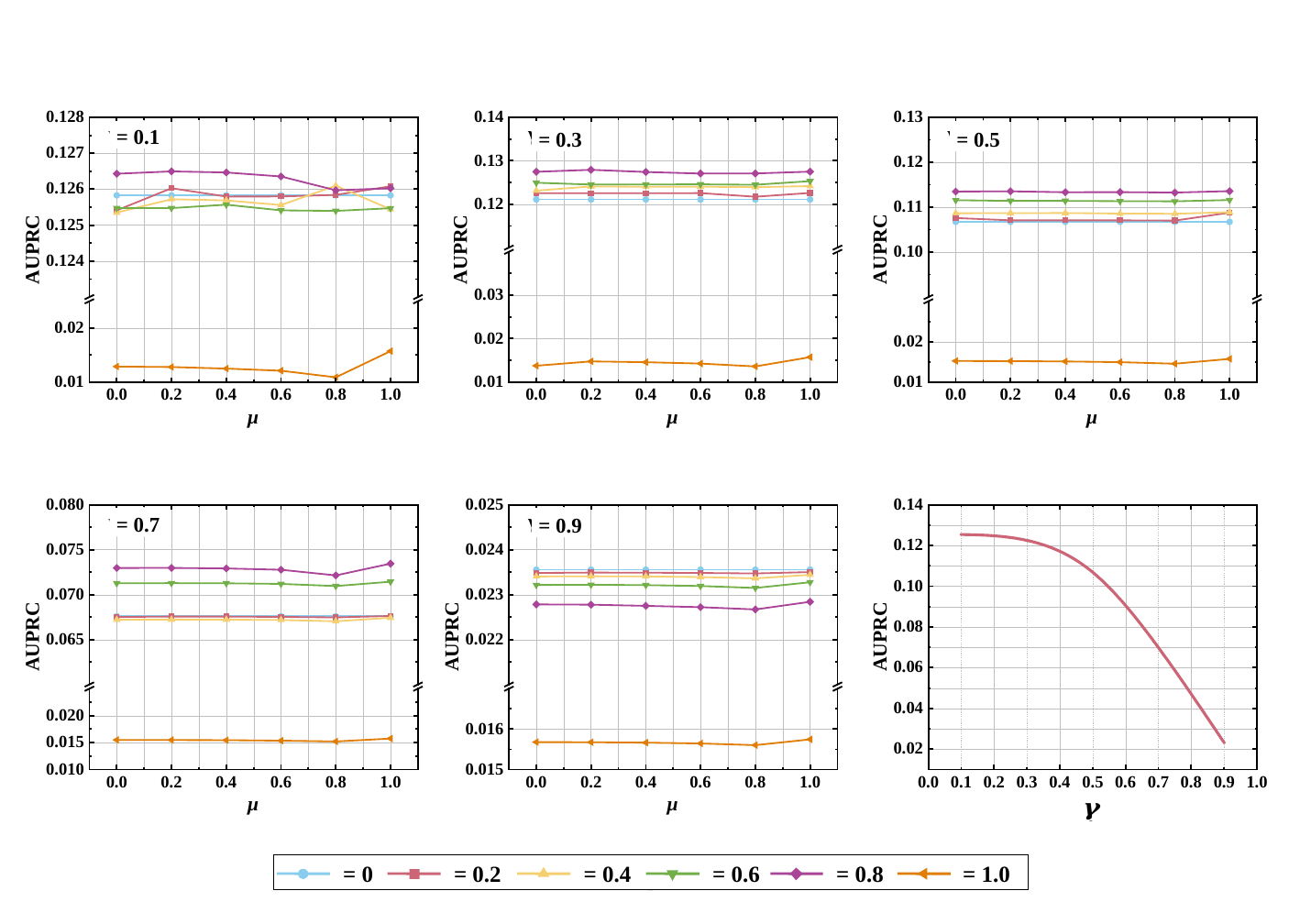

µ
1

Supplement: qzaf037_Supplementary_Data [file qzaf037_supplementary_data.zip › Figure S7.pptx]

## Slide 1
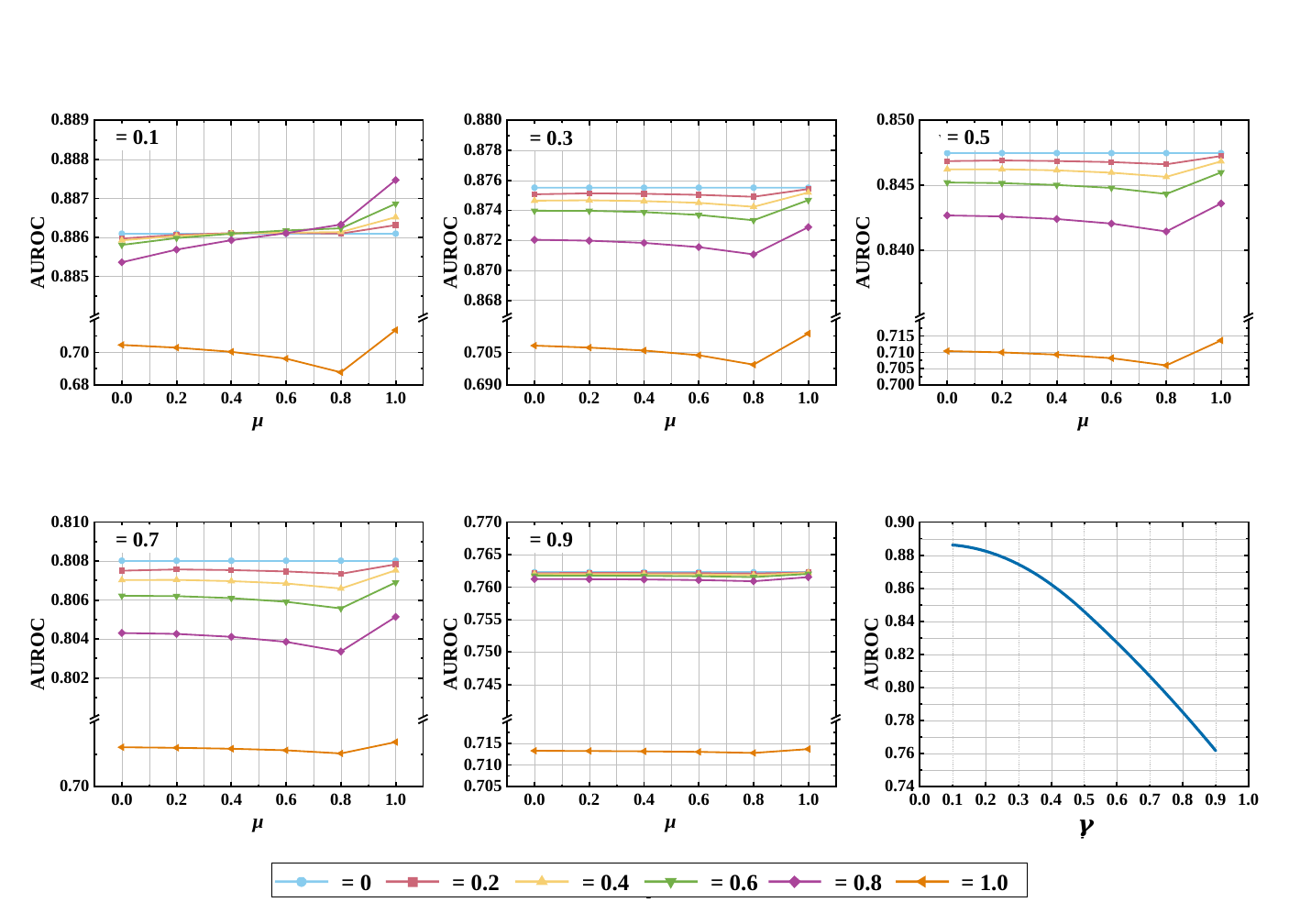

1
µ

Supplement: qzaf037_Supplementary_Data [file qzaf037_supplementary_data.zip › Figure S8.pptx]

## Slide 1
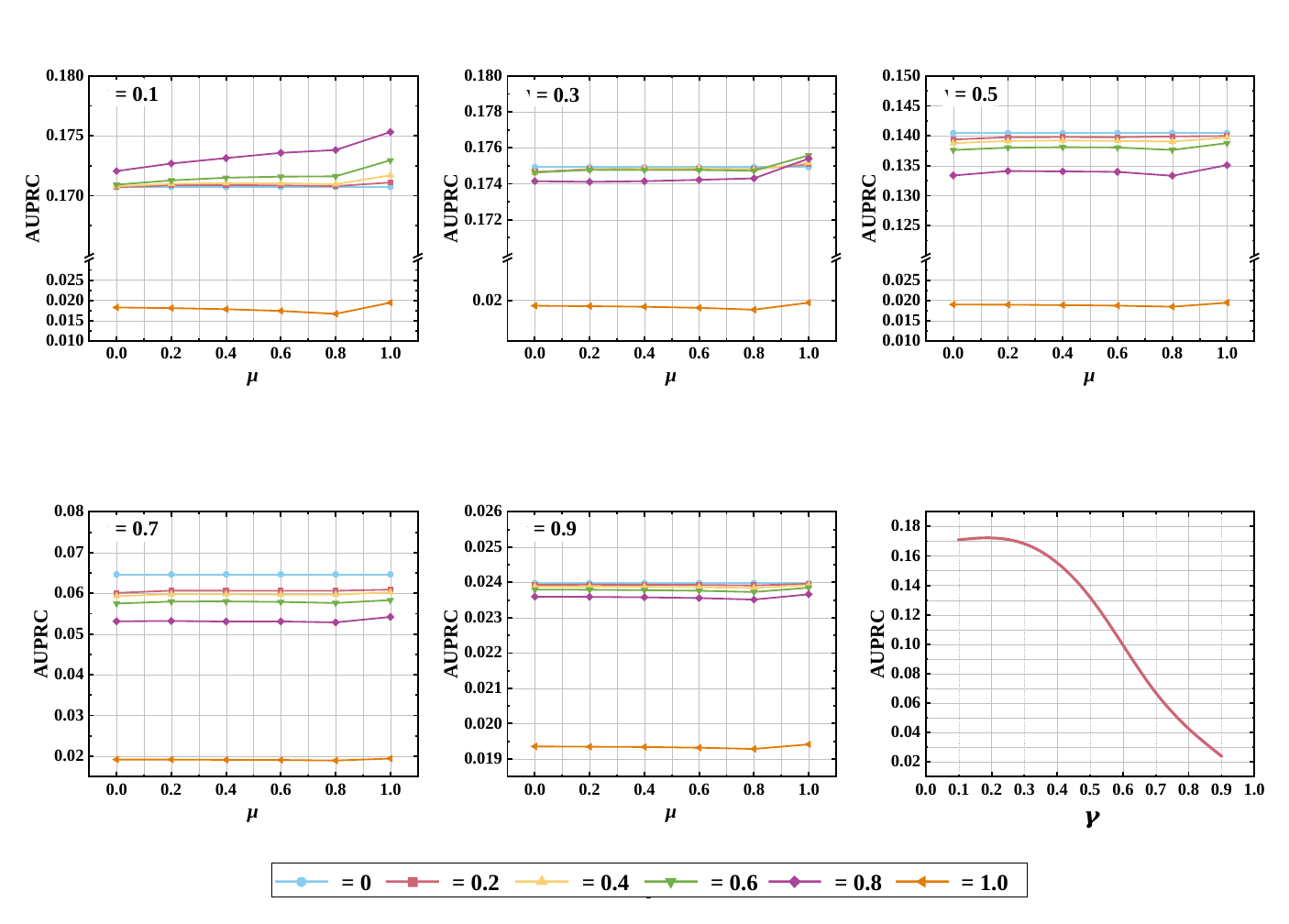

1
µ

Supplement: qzaf037_Supplementary_Data [file qzaf037_supplementary_data.zip › Figure S9.pptx]

## Slide 1
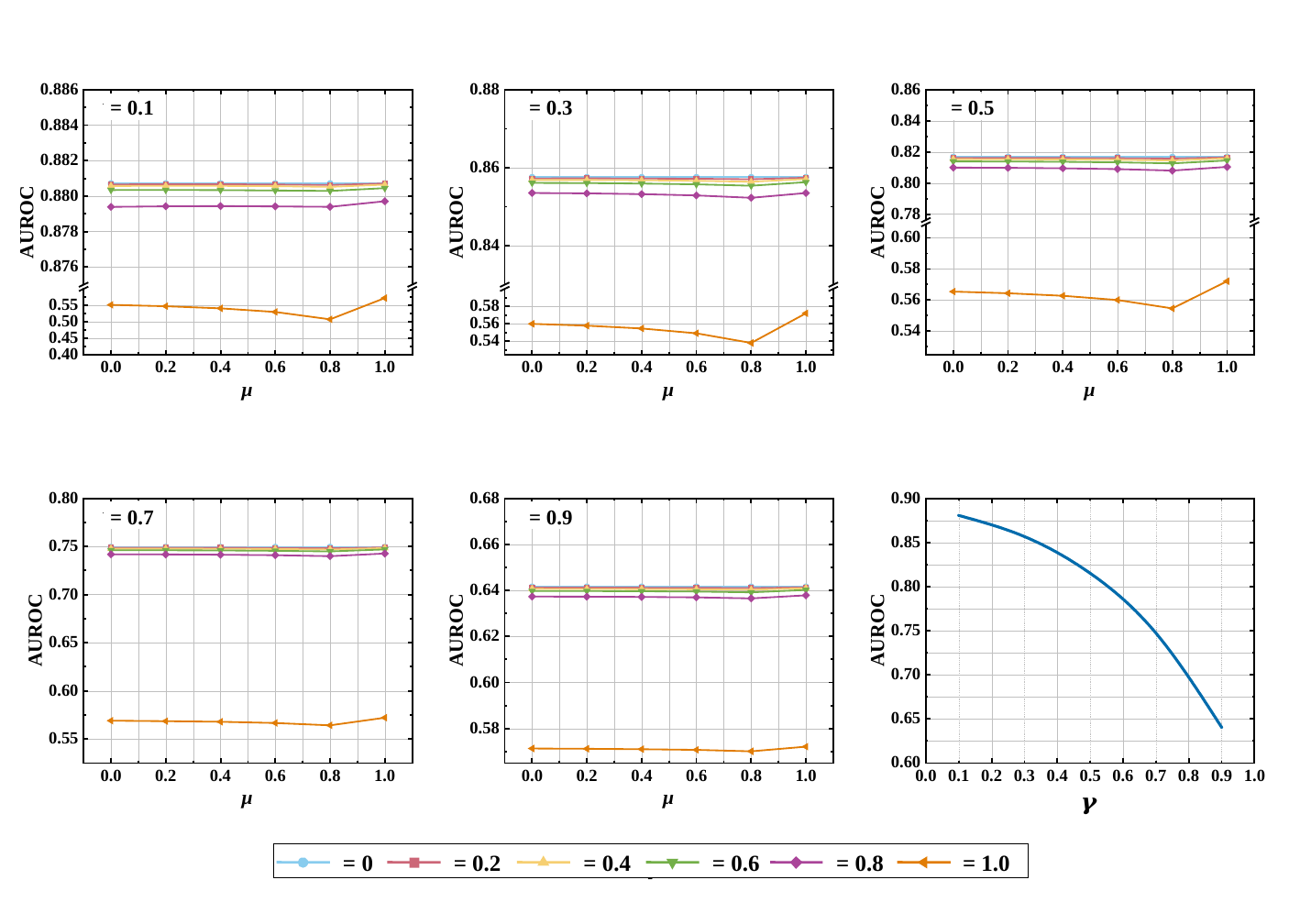

µ
1

Supplement: qzaf037_Supplementary_Data [file qzaf037_supplementary_data.zip › Figure S10.pptx]

## Slide 1
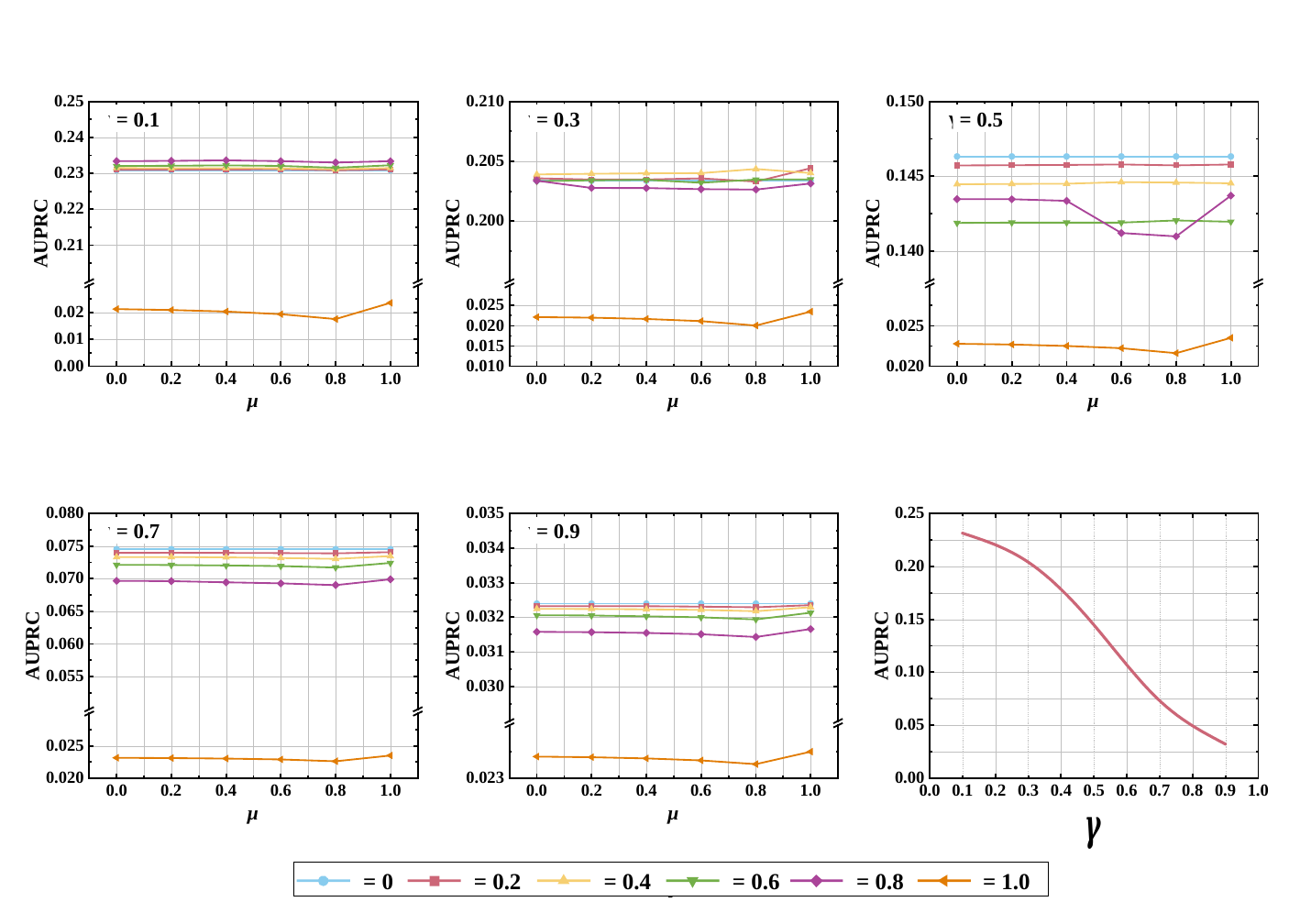

1
µ

Supplement: qzaf037_Supplementary_Data [file qzaf037_supplementary_data.zip › Figure S11.pptx]

## Slide 1
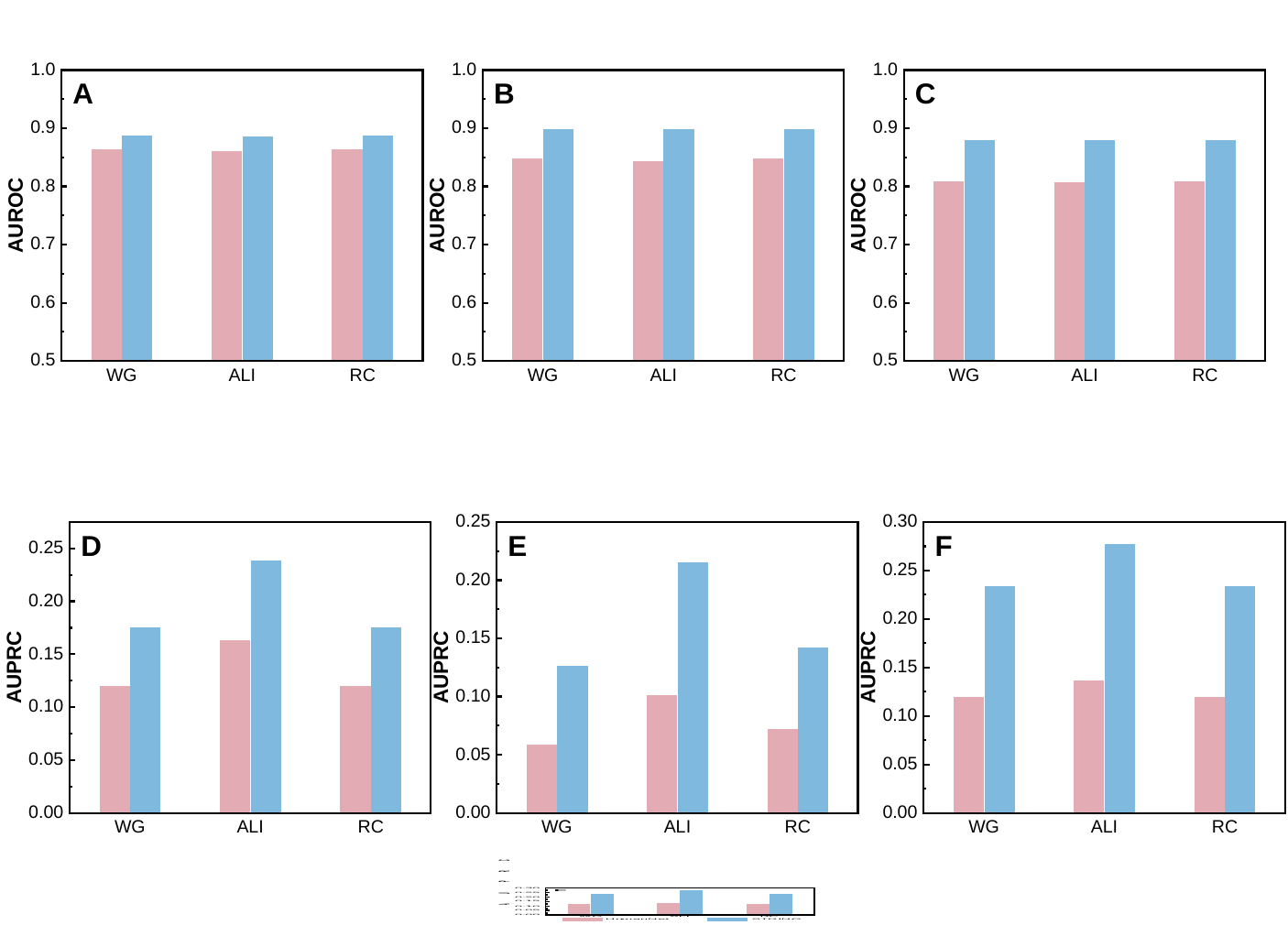

Supplement: qzaf037_Supplementary_Data [file qzaf037_supplementary_data.zip › Figure S12.pptx]

## Slide 1
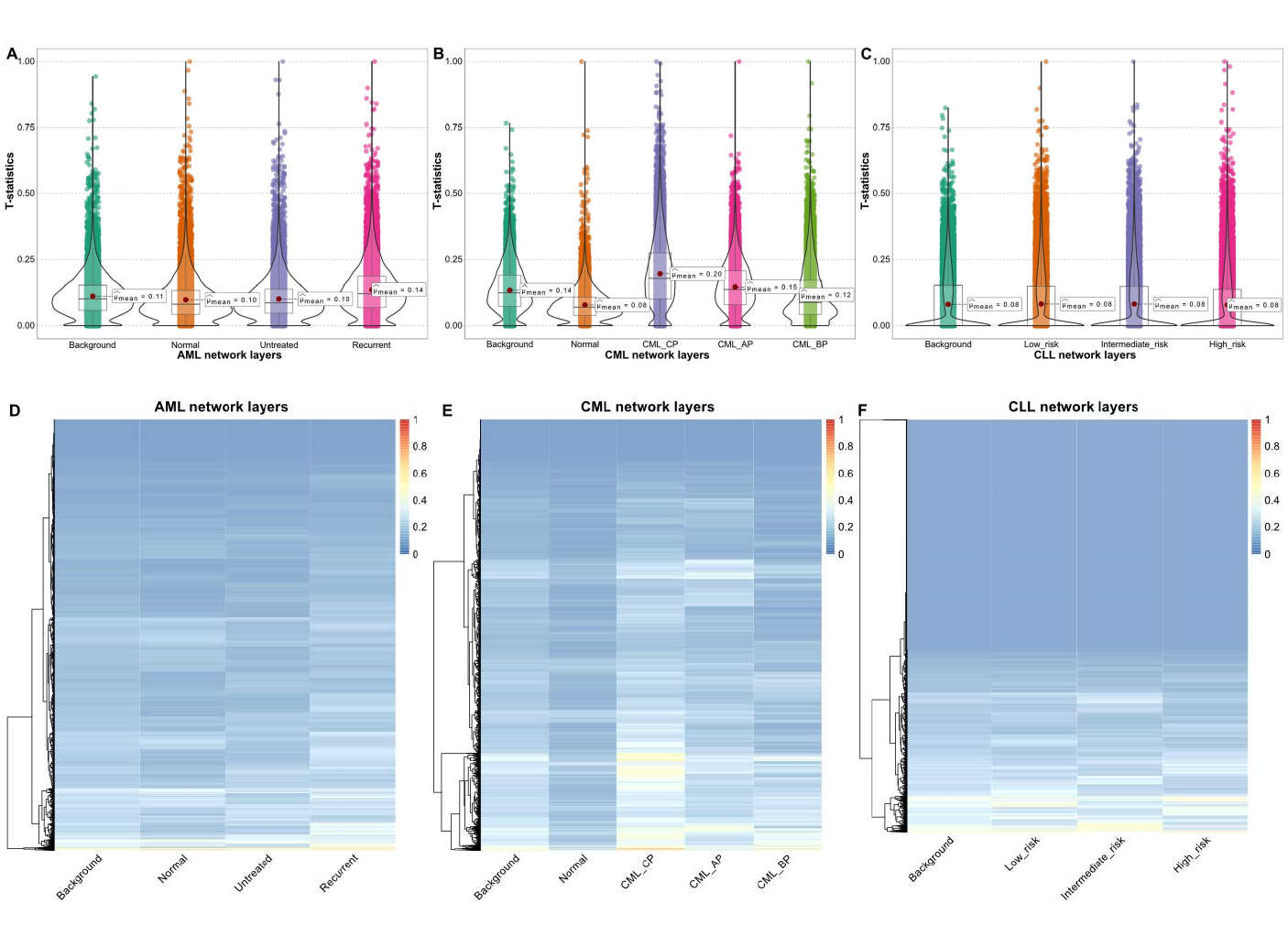

Supplement: qzaf037_Supplementary_Data [file qzaf037_supplementary_data.zip › Figure S13.pptx]

## Slide 1
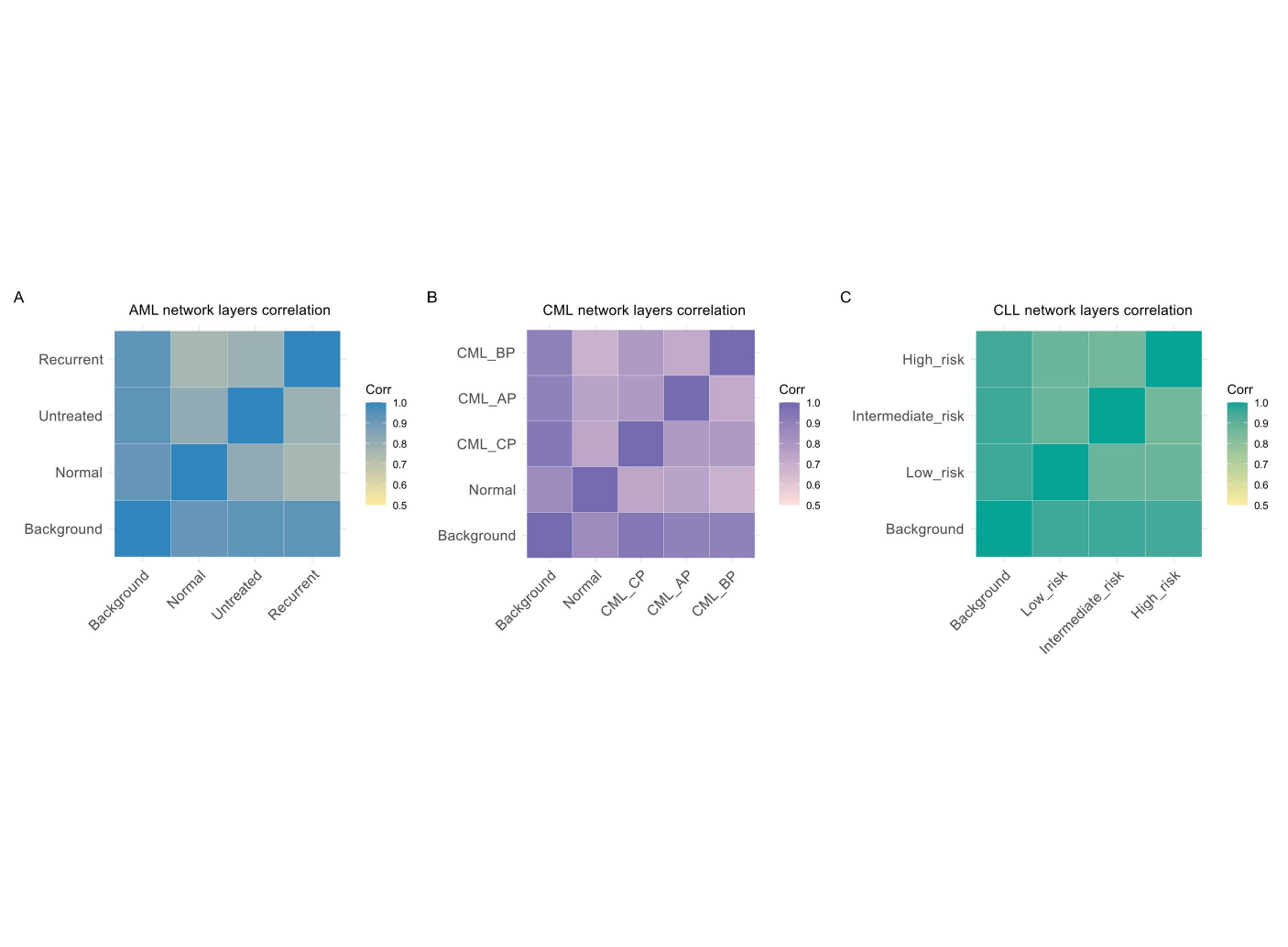

Supplement: qzaf037_Supplementary_Data [file qzaf037_supplementary_data.zip › Figure S14.pptx]

## Slide 1
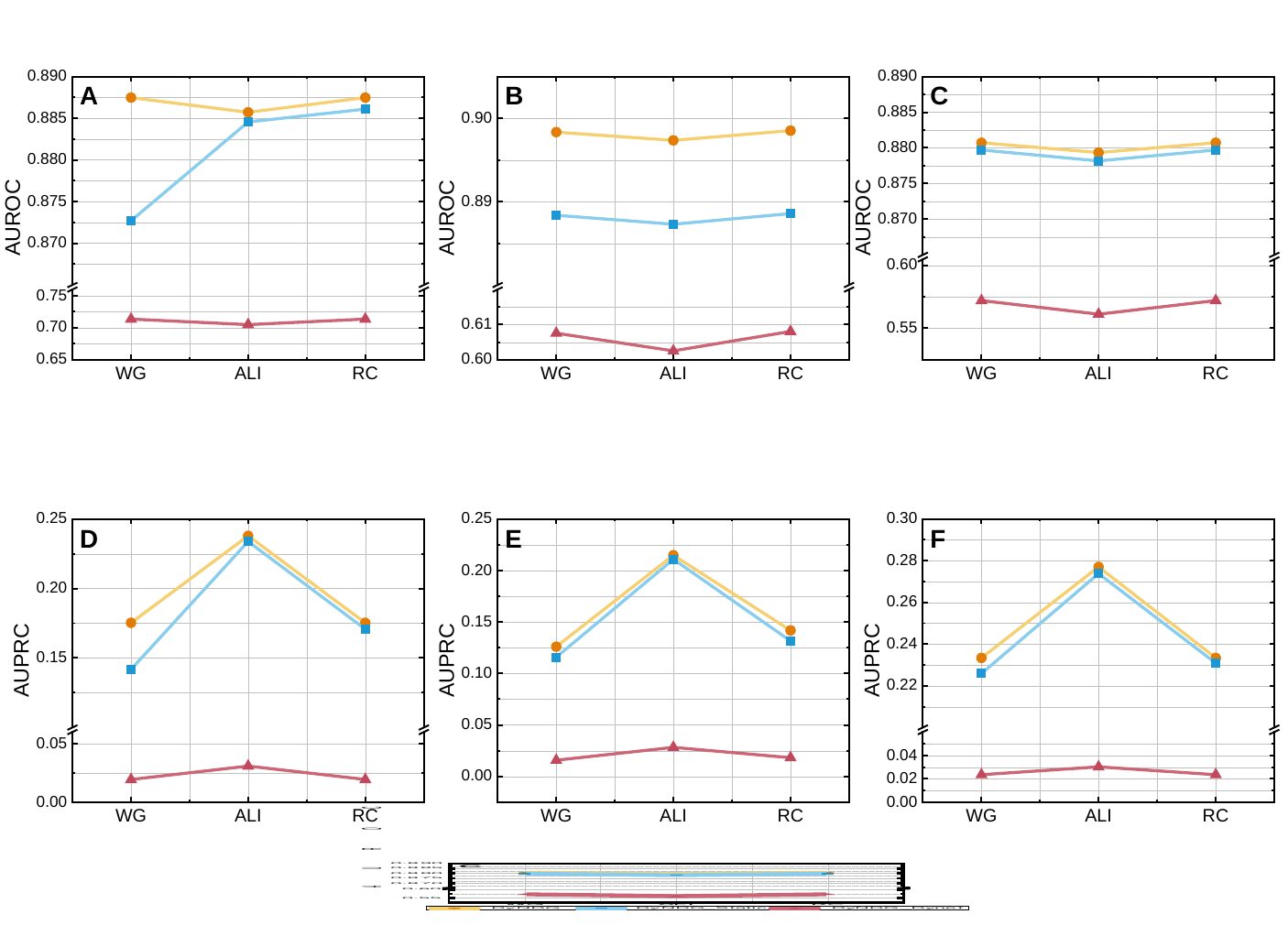

Supplement: qzaf037_Supplementary_Data [file qzaf037_supplementary_data.zip › Figure S15.pptx]

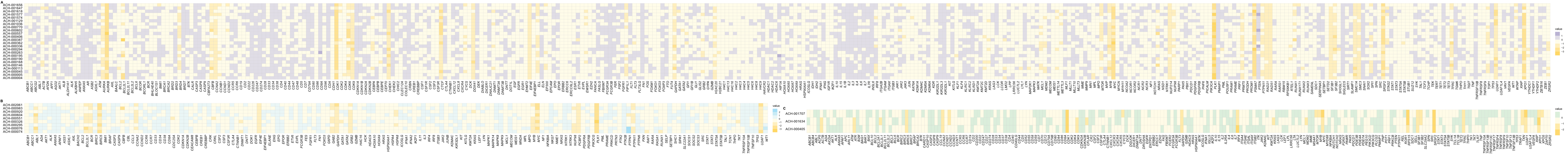

Supplement: qzaf037_Supplementary_Data [file qzaf037_supplementary_data.zip › Figure S16.jpg]
